# Histopathological, physiological and biochemical assessment of resveratrol nanocapsules efficacy in bleomycin-induced acute and chronic lung injury in rats

**DOI:** 10.1080/10717544.2022.2105445

**Published:** 2022-08-09

**Authors:** Neama M. Albanawany, Doaa M. Samy, Noha Zahran, Riham M. El-Moslemany, Shefaa Mf. Elsawy, Maha W. Abou Nazel

**Affiliations:** a Department of Histology and Cell Biology, Faculty of Medicine, Alexandria University, Alexandria, Egypt; b Department of Medical Physiology, Faculty of Medicine, Alexandria University, Alexandria, Egypt; c Department of Pharmaceutics, Faculty of Pharmacy, Alexandria University, Alexandria, Egypt

**Keywords:** Resveratrol nanocapsules, acute lung injury, fibrosis, inflammation and oxidative stress, ultrastructure

## Abstract

Acute lung injury (ALI) is a life-threatening illness which may progress to chronic pulmonary fibrosis (CPF). Resveratrol (RSV), a natural polyphenol, is known to exert several pharmacological effects on lung injury. However, its physicochemical properties and pharmacokinetic profile limit its clinical applications. In this study, RSV was loaded into lipid nanocapsules (LNCs) aiming to overcome these limitations. RSV-LNCs were prepared by phase inversion method and showed small uniform particle size (∼55 nm, PdI 0.04) with high entrapment efficiency >99%. The efficacy of RSV-LNCs in the prophylaxis against ALI and treatment of CPF was investigated in bleomycin-induced lung injury. For assessment of ALI, rats were administered a single oral dose of RSV (10 mg/kg) either free or as RSV-LNCs 4 h before bleomycin and euthanized 3 days later. For CPF, treatments in the same dose were given daily from days 10–20 after bleomycin and rats were euthanized on day-21. Results showed enhanced beneficial role for RSV-LNCs, compared to RSV, in the prevention of ALI as demonstrated by preservation of pulmonary microscopic and ultrastructural architecture and improvement of pulmonary functions. Analysis of BALF revealed reduction in oxidative stress markers, IL-6 level, leukocytosis and neutrophilia. iNOS and c-caspase 3 immunohistochemical expression and CD68^+^ cells immunofluorescence were inhibited. However, RSV-LNCs failed to show any improvement in oxidative stress, chronic inflammation, apoptosis and collagen deposition in CPF. In conclusion, RSV-LNCs are promising nanoplatforms for mitigating ALI detrimental effects. Future research investigating higher doses and longer durations of treatment is recommended to evaluate RSV-LNCs anti-fibrotic potential in CPF.

## Graphical Abstract

**Figure:**
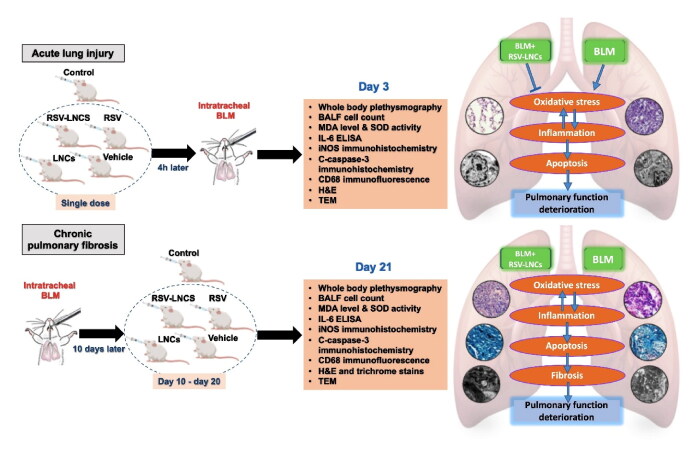

## Introduction

Acute respiratory distress syndrome (ARDS), a severe form of acute lung injury (ALI), is a major health concern due to associated morbidity (Meyerholz et al., 2018). Despite a falling death rate, it continues to be a significant contributing factor for mortality in critically ill patients (Mowery et al., 2020). ARDS can result either from direct pulmonary insult as in cases of bacterial and viral infections (e.g. COVID-19), aspiration and drug toxicity or indirect systemic inflammatory response as in cases of septicemia or shock (Fan & Fan, 2018).

ALI is characterized by infiltration with inflammatory cells and intense overwhelming secretion of cytokines (Fan & Fan, 2018; Zhou et al., 2018). This pro-inflammatory process may help remove the pathogenic stimuli. However, excessive inflammation in the lung may eventually progress to lethal cytokine storm (Aghasafari et al., 2019; Nile et al., 2020). Moreover, severe ALI triggers hypoxia with resultant establishment of inflammation-promoted pulmonary fibrosis (Shetty et al., 2017). Indeed, chronic pulmonary fibrosis (CPF) is a serious condition of progressive course and poor prognosis, for which no proven effective treatment till now (Shieh et al., 2019). Thus, addressing the pathological mechanisms that control local and systemic inflammatory reactions is crucial in the management of ALI (Panigrahy et al., 2020).

Bleomycin (BLM) is a chemotherapeutic agent that is used to treat some types of cancer. However, one of its major adverse effects is lung toxicity. In the first week following administration, BLM causes ALI via inducing DNA double-strand breaks, oxidative damage and release of pro-inflammatory cytokines. This acute response is followed by lung fibrosis in the next weeks. Intratracheal BLM injection is a well-established experimental model for assessing the pathogenic mechanisms involved in both acute and chronic pulmonary interstitial and airway diseases, and for evaluating relevant prospective therapeutics (Zhou et al., 2018; Humphries et al., 2021).

Traditionally, glucocorticoids have been used to dampen alveolar inflammation and prevent fatal cytokine storm. However, many drawbacks have been reported at high doses Further, glucocorticoids may even inhibit lymphocyte growth and depress immunity. As a result, researchers suggest that their use should be restricted to critical cases that require respiratory support. Thus, the necessity to explore comparatively safer natural alternatives is inevitable (Sharun et al., 2020; Solinas et al., 2020).

Resveratrol (RSV) is a natural polyphenol that belongs to the stilbenoid group found in grapes, peanuts, berries, etc. This polyphenolic compound has been shown to exert pleiotropic effects in many neurological (Griñán-Ferré et al., 2021), cardiac (Zivarpour et al., 2022) and pulmonary disorders (Li et al., 2018). However, RSV has an extremely short half-life, poor solubility and insufficient systemic delivery that reflect the need for high doses to display efficacy. Therefore, global research focus to minimize RSV’s physicochemical and pharmacokinetic limitations and optimize its systemic efficiency (Feng et al., 2017; Alanazi et al., 2020).

In this respect, nanotechnology-based strategies offer a great promise. The effect of RSV-loaded nanoparticles was studied in neurological, cardiac and metabolic disorders and reported promising results (Frozza et al., 2013; Mohseni et al., 2019; Zhang et al., 2019; Ashafaq et al., 2021). However, researches addressing nanotechnology-based approaches for increasing RSV effectiveness in pulmonary diseases are limited (de Oliveira et al., 2019, 2021).

Despite the success of nanoparticles in getting over the physicochemical and pharmacokinetic limitations of various drugs (Qiao et al., 2021), a challenging prospect for oral nanoparticle drug delivery is to overcome the several physiological barriers along the gastrointestinal tract, such as gastric acidity, intestinal mucus layer, and enzymatic degradation (Attama et al., 2012; Plaza-Oliver et al., 2021).

Lipid nanocapsules (LNCs) are relatively new nanovectors. They consist of a lipid core surrounded by lecithin and a pegylated surfactant rigid shell, thus providing stealth properties. LNCs are prepared by a low-energy solvent-free method (Heurtault et al., 2002). Their size ranges from 25 to 100 nm and are highly monodisperse (Heurtault et al., 2002). LNCs were shown to increase the bioavailability and efficacy of drugs following oral intake (Peltier et al., 2006; Ramadan et al., 2011; Ashour et al., 2020), which is the most convenient and economic route of administration. Successful oral drug delivery was attributed to their ability to maintain structural integrity in artificial gastrointestinal media (Roger et al., 2009) and in mucus (Groo et al., 2013). Moreover, several studies showed their ability to inhibit para-glycoprotein efflux and cross the intestinal barrier intact, thus allowing for targeting distant organs via the oral route (Amara et al., 2018).

In the current study, LNCs were prepared and characterized for their colloidal properties, morphology and RSV entrapment efficiency. Then, histological, physiological and biochemical investigations were used to determine the therapeutic potential of RSV-LNCs in BLM-induced lung disease. Two independent protocols for the use of RSV-LNCs were evaluated: first, the prophylactic potential for pretreatment with a single dose of RSV versus RSV-LNCs in ALI, and second, the anti-fibrotic effect of daily oral treatment with RSV or RSV-LNCs on established BLM-induced CPF.

## Materials and methods

### Materials

Resveratrol (RSV) was supplied by Ningbo Liwah Pharmaceutical Co., Ltd. (Zhejiang, China). Labrafac lipophile WL 1349 (caprylic-capric acid triglycerides, European Pharmacopeia, IVth, 2002) was a kind gift from Gattefossé S.A. (Saint-Priest, France). Kolliphor® HS 15 (a mixture of free polyethylene glycol 660 and polyethylene glycol 660 hydroxystearate, European Pharmacopeia, IVth, 2002) was provided by BASF (Ludwigshafen, Germany). Oleic acid (OA) was purchased from Sigma Aldrich (USA). Bleomycin (BLM) was purchased in the form of commercial Bleocel^®^ 15 IU Injection manufactured by celon Lab.

### Preparation of lipid nanocapsules

Lipid nanocapsules (LNCs) were prepared by the phase inversion method which entails heating/cooling cycles from 65–85 °C (Heurtault et al., 2002). Since RSV stability is highly affected by light and temperature (Zupancic et al., 2015), preparation conditions were tightly controlled, and oleic acid was added to the formulation as it is capable of reducing the phase inversion temperature (38 °C) (Eissa et al., 2015).

Briefly, Kolliphor, Labrafac, deionized water (1:1:3) were weighed and mixed under magnetic stirring. Oleic acid (OA) and sodium chloride (1.5 and 0.44% w/v of the final volume, respectively) were then added to the mixture and subjected to three heating/cooling cycles between 35 and 50 °C. At the phase inversion temperature, an irreversible shock by 4-fold dilution with deionized cold water (0–2 °C) was induced. Following quenching, slow magnetic stirring of the LNCs dispersion continued for 5 min. For RSV-LNCs, the drug was added from the beginning of the formulation process at concentration 2 mg/ml of the final dispersion volume. The formulation was protected from light throughout the process to avoid drug decomposition.

### Physicochemical characterization of the formulation

#### Colloidal properties

The average particle size and polydispersity index (PdI) of LNCs formulations were determined by dynamic light scattering (DLS) using Malvern Zetasizer^®^ at a fixed angle (173°) at 25 °C using a 4mW He-Ne laser at 633 nm (Zetasizer^®^ Nano ZS series DTS 1060, Malvern Instruments S.A, Worcestershire, UK). Zeta potential was determined at 25 °C in water (dielectric constant 79, refractive index 1.32, viscosity 0.88 cP) using a cell voltage of 150 V and 5 mA current. LNCs dispersions were appropriately diluted with filtered deionized water before measurement. Analyses were performed in triplicate.

#### Morphology

LNCs morphology was examined by transmission electron microscopy (TEM, JEM-100 CX, JEOL, Japan) where LNCs dispersion was four folds diluted and sprayed onto copper grids and stained with 2% w/v uranyl acetate solution then shots taken at × 50 K at 80 kV.

#### Entrapment efficiency

Entrapment efficiency (EE%) was calculated using the ultrafiltration/centrifugation technique (Sigma 3-30KS, Sigma Laborzentrifugen GmbH, Germany) by determining free resveratrol (un-entrapped) concentration in the ultrafiltrate spectrophotometrically. LNCs dispersion was added to the ultracentrifugal concentrator (Sartorius™ Vivaspin6™, MWCO 100,000) and centrifuged for 15 min at 3663 × g then absorbance measured at 324 nm. RSV concentration was calculated using calibration standards. Linearity was checked within the 0.5–4 μg/ml. Measurements were done in triplicate. The concentration of RSV entrapped was calculated using the following equation:

$$\text{EE}\;\%\;=\;\frac{\mathrm{Total}\;\mathrm{drug}\;\mathrm{(mg)–unentrapped}\;\mathrm{drug}\;\mathrm{(mg)}}{\mathrm{Total}\;\mathrm{drug}\;\mathrm{(mg)}}\times \;100$$

#### In vitro drug release

RSV-LNCs were 10-fold diluted with phosphate buffer pH 7.4 containing 0.5% tween 80 to maintain sink conditions. Samples were placed at 37 °C at 100 rpm in a thermostatically controlled shaking water bath. At predetermined time intervals, LNCs were separated using ultracentrifugal concentrators and the filtrate analyzed spectrophotometrically at 324 nm. The % RSV released was calculated in triplicate relative to the theoretical initial drug content and release profile (percent release versus time) plotted.

### Animals and study design

Eighty adult male albino rats (200–220 g) were acclimatized for 2 weeks before the start of the experiment under a 14:10 h light: dark cycle and supplied with food and water ad libitum. Experiments were conducted in accordance with the national guidelines that comply with the ARRIVE guidelines for the care and use of laboratory animals. Ethical approval was obtained from the Medical Ethics Committee of Alexandria Faculty of Medicine (IRB NO: 00012098-FWA NO: 00018699-approval number: 0201451).

Pulmonary injury was induced by a single intratracheal instillation of BLM (5 mg/Kg dissolved in 0.4 ml PBS) (Liu et al., 2016). Rats were randomly assigned to one of 2 paradigms according to the time elapsing between BLM administration and study termination: 3-day and 21-day paradigms, for assessment of ALI and CPF, respectively. In each paradigm, a cohort of 32 rats was subdivided into 4 groups (8 rats each): **BLM + vehicle** (received intratracheal BLM and treated orally with saline), **BLM + LNCs** (received intratracheal BLM and treated with blank LNCs dispersion), **BLM + RSV** (received intratracheal BLM and treated with 10 mg/kg RSV, prepared by adding the drug (2 mg/ml) to a solution of PEG 400: Propylene glycol: saline at concentrations 1:1:8 by volume) and **BLM + RSV-LNCs** (received intratracheal BLM and treated with RSV-LNCs equivalent to 10 mg/kg RSV) (de Oliveira et al., 2019).

In the ALI paradigm, each rat received a single dose of treatment 4 hours prior to BLM administration by oral gavage and then euthanized on the 3rd day following BLM administration (Zhou et al., 2018). For the CPF paradigm, animal subgroups were treated by daily oral gavage for 11 days starting from day 10 following BLM administration and then euthanized on the 21st day (Baek et al., 2020). Additional **control** group (*n* = 8) for each cohort received intratracheal PBS and treated orally with vehicle for the same protocol period.

### Pulmonary function assessment

Lung function was measured in conscious and unrestrained animals using whole body plethysmography (WBP) before BLM administration and at the end of experimental period. For this purpose, rats were placed inside a sealed acrylic plethysmographic chamber, and changes in air pressure inside the chamber during inspiration and expiration were detected using a differential pressure transducer connected to a pneumotach flow head (MLT1L, spirometer; ADInstruments). A PowerLab digital system (4/25, ADInstrument, Bella Vista, Australia) with LabChart 8 software (ADInstruments, Castle Hill, NSW, Australia) was used for data acquisition. The temperature inside and outside the chamber was continuously monitored. The pulmonary function of each rat was recorded while the animal was stationary and calm for 3 minutes (Rodrigues et al., 2021; Zakaria et al., 2021).

The ventilatory parameters calculated include tidal volume (TV; ml), Breathing frequency (BF; bpm), minute respiratory volume (MRV; ml/min), inspiration time (Ti; s), expiration time (Te; s), total breathing cycle time (Ttot; s), inspiratory duty cycle (Ti/Ttot; %) and inspiratory flow rate (VT/Ti; ml/s). The enhanced pause (Penh) was also calculated using the following formula:

Penh=(TeTr−1)(PEFPIF)

where Tr is the relaxation time (s), PIF is the peak inspiratory flow (ml/s), and PEF is the peak expiratory flow (ml/s) (Hamelmann et al., 1997; Lan et al., 2015). The Penh is an index for airflow during the respiratory cycle, and positively correlates with airway resistance or forced expiration (Hülsmann et al., 2021). The Penh values of spontaneously breathing mice were measured within a 3-min period and average values were calculated.

### Bronchoalveolar lavage fluid analysis

At the end of experiment, all rats were euthanized and bronchoalveolar lavage was performed twice using an airway catheter inserted in the rat’s trachea under sterile conditions. In each time, 5 ml of saline was infused into the airway, then bronchoalveolar lavage fluid (BALF) was recovered. BALF samples underwent centrifugation at 500 × g for 10 min at 4 °C, and supernatants were collected and stored at −20 °C for assessment of the level of the proinflammatory cytokine IL-6 using enzyme-linked immunosorbent assay (ELISA) kits (#201-12-0091 SunRed), and oxidative stress markers (SOD activity and MDA level) using colorimetric technique.

The pellets were resuspended in 50 μl PBS to determine total and differential white blood cell count using an automated hemocytometer in a double-blind manner.

### Histological examination of the lung

Both lungs were immediately dissected and each side was divided into two parts and processed for the following histological examinations:

- **Light microscopic examination**

The first part of lung tissue was cut in pieces of 0.5 mm thickness, fixed in 10% formol saline and processed to get 5 μm thick serial paraffin sections. These sections were stained with hematoxylin-eosin (H&E) or modified Masson’s trichrome stains. Histological sections were examined using light microscope (Olympus BX41) equipped with spot digital camera (Olympus DP20). Histomorphometric analysis for inter-alveolar septal thickness (μm) and alveolar surface area (μm^2^) of H&E stained sections and area percentage (%) of deposited collagen in Masson’s trichrome stained sections was performed using NIH Fiji program (NIH, Bethesda, MD, USA).

- **Immunohisto-staining**

For immunohistochemical study, serial lung sections obtained from the paraffin blocks (after antigens recovery) were reacted with the following primary antibodies: rabbit polyclonal anti-inducible nitric oxide synthase (iNOS; 1:500; ab178945) to assess inflammation and rabbit polyclonal anti-cleaved caspase-3 (c-caspase 3; 1:1000; #9662; Cell Signaling Technology) to assess apoptosis. Briefly, paraffin-embedded sections (thickness, 5 μm) were deparaffinized, treated with 3% H_2_O_2_ in methanol for 10 min to inactivate any endogenous peroxidase, and then incubated for 30 min in blocking buffer (5% bovine serum albumin in Tris-buffered saline with Tween 20 [TBST]). The sections were incubated overnight at 4 °C with the primary antibodies, followed by incubation at (20–25 °C) temperature with an HRP-conjugated secondary antibody (1:100; Sigma-Aldrich, USA) diluted in 1% BSA. Sections were examined using light microscope. Integrated optical density (IOD) of immune-reactivity was measured in arbitrary units (A.U.) using NIH Fiji program (NIH, Bethesda, MD, USA).

- **Immunofluorescence staining**

For immunofluorescence detection of CD68^+^ (a macrophage marker), serial lung sections from the paraffin blocks were incubated with rabbit polyclonal Anti-CD68^+^ antibody (1/100 in PBS; ab125212). The sections were incubated overnight at 4 °C with the primary antibody, followed by incubation for 1 h at room temperature with fluorescent dye conjugated Goat anti-rabbit IgG polyclonal secondary antibody (1/200). Number of CD68^+^ cells and mean fluorescence intensity (expressed in A.U.) in each lung field were determined using NIH Fiji program (NIH, Bethesda, MD, USA).

- **Transmission electron microscopic examination**

The second part of lung tissue was immediately cut into small pieces (1/2–1 mm^3^), fixed in 3% glutaraldehyde solution and processed to get ultrathin sections that were examined using TEM (JEM-100 CX Electron Microscope, JEOL, Tokyo, Japan).

### Statistical analysis

All results were statistically analyzed using the statistical package for the Social Sciences (SPSS) version 22, and graphical representations were performed with GraphPad Prism 7 software. Shapiro-Wilk’s test was used to verify the normality of distribution. Data were presented as the means ± standard deviation (S.D.). Significant differences between groups of the same cohort were compared using one-way ANOVA with post hoc Tukey’s test for pairwise comparison, while difference of data between acute (day 3) and chronic (day 21) BLM + vehicle groups was analyzed by unpaired t-test. The p value was judged at the 5% level.

## Results

### Characterization of nanocapsules

**Figure 1.**
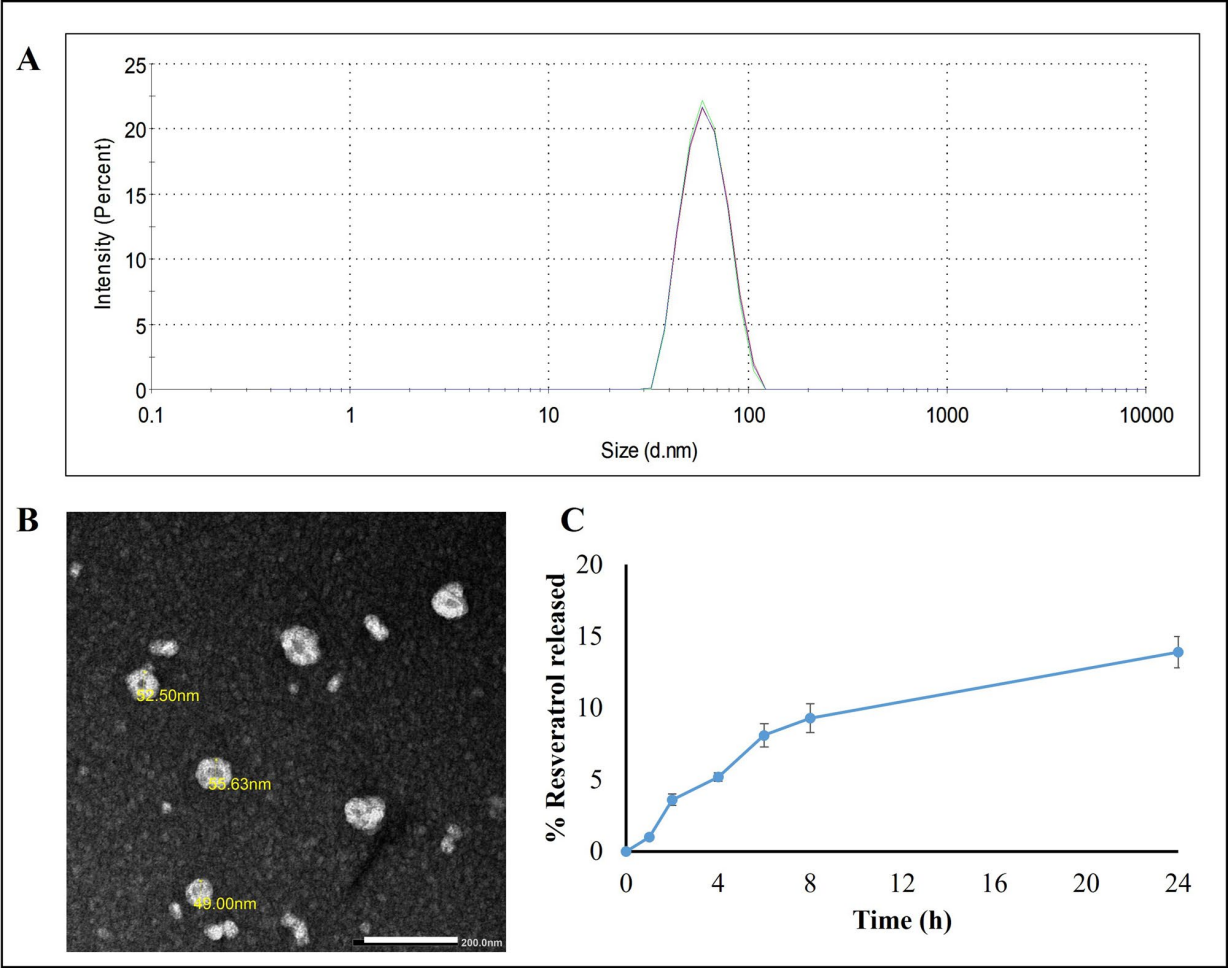
(A) Size distribution by intensity curve of RSV-LNCs dispersion. (B) TEM image of RSV-LNCs × 40,000. The scale bar represents 200 nm. (C) Resveratrol release profile from LNCs over 24 h at 37 °C (*n* = 3; data are shown as mean ± SD).

LNCs showed a particle size 54.5 ± 0.2 nm with a slightly negative zeta potential −7.72 ± 1.32 mV previously attributed to the presence of small proportion of hydrolyzed surfactants (Eldesouky et al., 2021). A PdI of 0.04 indicated monodispersity of the formulation (Figure 1A). RSV loading did not affect colloidal properties. TEM imaging of RSV-LNCs is shown in (Figure 1B). LNCs were spherical with homogenous distribution and were not aggregated.

EE% of RSV in LNCs was measured spectrophotometrically and was shown to exceed 99%. The high EE is due to the drug lipophilicity which allows its incorporation in the oily core (Eldesouky et al., 2021).

*In vitro* drug release was studied over 24 h (Figure 1C). RSV-LNCs showed a typical sustained and prolonged drug release profile with only 14% RSV released over 24 h. This ensures successful encapsulation of RSV in the oily core and is in accordance with previously reported lipophilic drug release patterns from LNC formulations (Roger et al., 2011; Eissa et al., 2015; Amara et al., 2018).

### Effect of RSV-LNCs on BLM-induced deterioration in pulmonary functions

**Figure 2.**
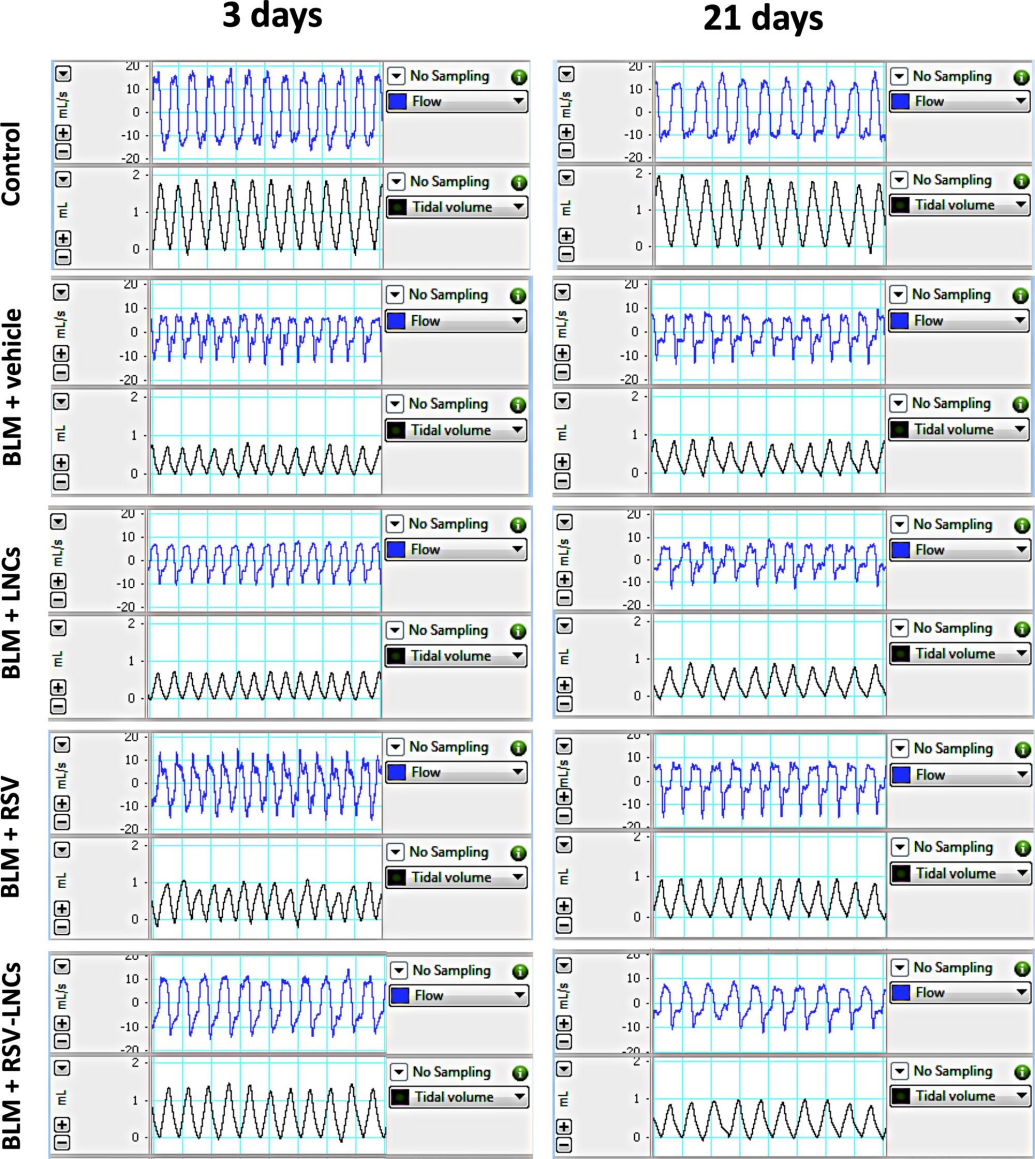
Representative tracings of airway flow and tidal volume in the studied groups performed by whole body plethysmography at day 3 and day 21 following bleomycin (BLM) instillation.

WBP was used to monitor pulmonary functions of the treated rats (Figure 2). At day 0, no significant difference was observed between experimental groups (all *p* > 0.05) (data not shown). Three days after BLM administration, rats showed an increase in respiratory rate by 31.21% (*p* < .001). By day 21, the respiratory rate returned to within basal levels (*p* > 0.05). The V_T_, MRV and inspiratory flow rate of BLM intoxicated rats decreased by 56.36%, 42.74% and 56.67% on day 3 (all *p* < .001), and by 50.83%, 43.94% and 44.82% on day 21 (all *p* < 0.001), respectively compared to corresponding control rats. Moreover, rats were assessed for airway dysfunction by measuring Penh. The Penh reflects changes in the inspiratory and expiratory flow, and combines these changes with the timing of early and late expiration. Intratracheal BLM produced approximately a three-fold and a six-fold increment in the Penh at day 3 and day 21, respectively, after BLM administration compared with control rats (both *p* < .001). LNCs failed to revert BLM-induced acute derangements (all *p* > 0.05).

The rats pretreated with RSV showed mild remission in pulmonary functions at day 3, where Penh decreased by 39.34% (*p* = .032), while MRV increased by 29.4% (*p* = .017), without a significant effect on BLM-induced tachypnea, V_T_ and inspiratory flow rate (all *p* > 0.05).

RSV-LNCs pretreatment successfully mitigated pulmonary functions at day 3 after BLM instillation as revealed by decreased BF by 17.18% (*p* = .006), increased V_T_ by 91.67% (*p* < .001), increased MRV by 58.74% (*p* < .001) and increased inspiratory flow rate by 72.55% (*p* = .035), compared to acute BLM group. in addition, RSV-LNCs caused a 60.03% decrease (*p* < .001) in Penh value after 3 days of BLM instillation (Table 1).

**Table 1.** Assessment of pulmonary functions using whole body plethysmography at day 3 and day 21 after bleomycin (BLM) administration.

			BLM			
		Control	Vehicle	LNCs	RSV	RSV-LNCs
V_T_ (ml)	3 days	1.65 ± 0.34	0.72 ± 0.21**	0.71 ± 0.39**	1.00 ± 0.11**	1.40 ± 0.18^##€^
	21 days	1.81 ± 0.34	0.89 ± 0.37**	0.83 ± 0.26**	0.93 ± 0.33**	0.95 ± 0.46**
MRV (ml/min)	3 days	285 ± 30	163 ± 17.5**	155 ± 27.3**	211 ± 31.4**^#^	259 ± 35.2^##€^
	21 days	284 ± 18.3	159 ± 15.8**	148 ± 30.2**	164 ± 27.2**	166 ± 20**
Breathing rate (bpm)	3 days	173 ± 16.1	227 ± 22.4**	219 ± 28.5**	211 ± 15.2*	188 ± 20.7^#^
	21 days	157 ± 15.3	179 ± 20.5^¥¥^	167 ± 21.3	175 ± 28.1	177 ± 29.8
Inspiratory flow rate (ml/s)	3 days	11.62 ± 2.6	5.03 ± 2.64**	5.46 ± 0.95**	7.63 ± 1.5*	8.68 ± 3.48^#^
	21 days	10.34 ± 2.86	5.71 ± 1.35**	5.22 ± 1.28**	5.74 ± 2.05*	6.29 ± 2.56*
Inspiratory duty cycle (%)	3 days	44.8 ± 10.6	54.2 ± 9.5	48.2 ± 16.8	48.4 ± 10.5	49.4 ± 10.6
	21 days	46.2 ± 22	47 ± 12.6	41.8 ± 16.3	49.4 ± 5	44.4 ± 10.4
Penh	3 days	0.66 ± 0.29	1.95 ± 0.42**	1.6 ± 0.59**	1.32 ± 0.29*^#^	0.71 ± 0.4^##€^
	21 days	0.48 ± 0.31	2.74 ± 0.64^¥^**	2.66 ± 0.56**	2.68 ± 0.37**	2.29 ± 0.78**

LNCs: unloaded lipid nanocapsules; RSV: resveratrol; RSV-LNCs: resveratrol-loaded lipid nanocapsules; V_T_: tidal volume; MRV: minute respiratory volume; Penh: enhanced pause. The data are presented as the means ± SD (*n* = 8). Difference between groups at the same time point was analyzed by one-way ANOVA followed, when significant, by Tukey post-hoc test. *p < .05, **p ≤ .001 compared with control; ^#^*p* < . 05, ^##^*p* ≤ .001 compared with BLM + vehicle group; ^€^*p* < .05, ^€€^*p* ≤ .001 compared with BLM + RSV group. Difference of data between acute (day 3) and chronic (day 21) BLM + vehicle groups was analyzed by unpaired t-test, where ^¥^*p* < .05, ^¥¥^*p* ≤ .001.

On the other hand, at day 21 after BLM challenge, neither treatment could ameliorate the Penh index or other lung functions, as no significant difference was detected in the tested parameters compared to the BLM group (all *p* > 0.05) (Table 1).

### Effect of RSV-LNCs on BLM-induced pulmonary oxidative stress

**Figure 3.**
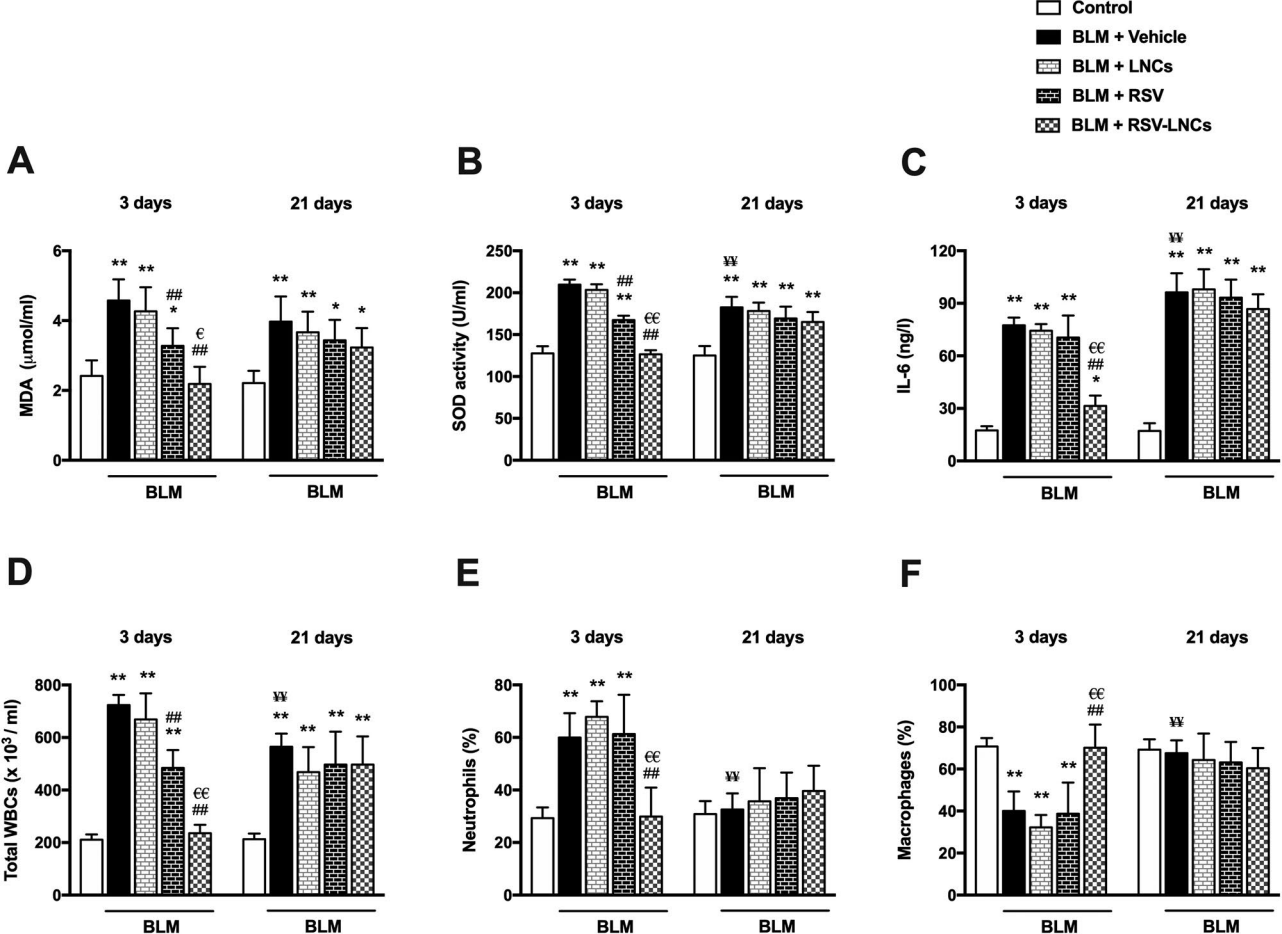
Effect of RSV-LNCs on BLM-induced pulmonary oxidative stress, IL-6 levels and leukocytic count in BALF. (A, B) Oxidative stress biomarkers: malondialdehyde (MDA) and superoxide dismutase (SOD) activity. (C) IL-6 levels in BALF measured by ELISA. (D–F) Total and differential WBCs count detected by an automated hemocytometer. Data are presented as the means ± S.D. (*n* = 8/group). Difference between groups at the same time point was analyzed by one-way ANOVA followed, when significant, by Tukey post-hoc test. *p < .05, **p ≤ .001 compared with control; ^#^*p* < . 05, ^##^*p* ≤ .001 compared with BLM + vehicle group; ^€^*p* < .05, ^€€^*p* ≤ .001 compared with BLM + RSV group. Difference of data between acute (day 3) and chronic (day 21) BLM + vehicle groups was analyzed by unpaired t-test, where ^¥^*p* < .05, ^¥¥^*p* ≤ .001.

SOD activity and MDA level in BALF were used to assess oxidative lung injury. As demonstrated in Figure 3(A) and 3(B), BLM toxicity in the acute phase was associated with increased MDA along with enhanced SOD activity (both *p* < .001) relative to control values. These oxidative stress markers were normalized by RSV-LNCs pretreatment (*p* > 0.05 versus control group). RSV pretreatment also reduced BLM-induced oxidative stress (*p* < .001 versus BLM group), but significantly less than RSV-LNCs (*p* < .001 and *p* = .003 for SOD activity and MDA level, respectively), meanwhile LNCs showed no significant effect on either SOD activity or MDA level (all *p* > 0.05).

On day 21, BLM-induced oxidative stress was evident by high MDA level and SOD activity in BALF (both *p* < .001) compared to the control group, although SOD activity significantly decreased compared to the acute phase (*p* < .001). All treatments could not mitigate these oxidative stress markers, as no significant change was observed in rats treated with either LNCs, RSV or RSV-LNCs compared with the BLM group (all *p* > 0.05) (Figure 3A and 3B).

### Effect of RSV-LNCs on IL-6 in BALF

The level of the pro-inflammatory and fibrogenic cytokine, IL-6, was measured in BALF. At the third day following BLM administration, the levels of IL-6 in the animals received LNCs and RSV were comparable to the untreated group (*p* > 0.05), which was significantly higher than that of the control group (*p* < .001). However, oral pretreatment with RSV-LNCs significantly attenuated IL-6 levels versus BLM group (*p* < .001).

Three weeks after BLM challenge, the level of IL-6 was further increased compared with acute levels (*p* = .001) and matched control levels (*p* < .001). Treatment with LNCs, RSV or RSV-LNCs did not show any effect on IL-6 levels in the fibrotic phase (all *p* > .05) (Figure 3C).

### Effect of RSV-LNCs on inflammatory cells in BALF

In BALF recovered at day 3, BLM toxicity induced a significant increase in total leukocyte count associated with an increase in neutrophils percent and a parallel decrease in macrophages percent (*p* < .001), compared to the control levels. LNCs administration did not affect the leukocytic profile compared to the BLM group (*p* > 0.05). Although rats received free RSV exhibited a significant decrease in total leukocytes count (*p* < .001), cell differentiation analysis was not significantly altered (*p* > 0.05), compared to the BLM group. The BLM-induced leukocytic infiltration was completely suppressed by RSV-LNCs pretreatment (*p* < .001 versus BLM group), with shift toward control percentages of neutrophils and macrophages (Figure 3D–F).

At day 21, the total leukocyte counts in BALF of BLM-intoxicated group decreased as compared to day 3 (*p* < .001), however, it was higher than values in matched controls (*p* < .001). This inflammatory response was not alleviated by any treatment received, as all treated groups had total leukocytic count comparable to the BLM group (all *p* > 0.05). Moreover, no significant difference was elucidated among the experimental groups in the neutrophils percent or the macrophages percent (all *p* > 0.05) (Figure 3D–F).

### Effect of RSV-LNCs on BLM-induced pulmonary tissue injury

**Figure 4.**
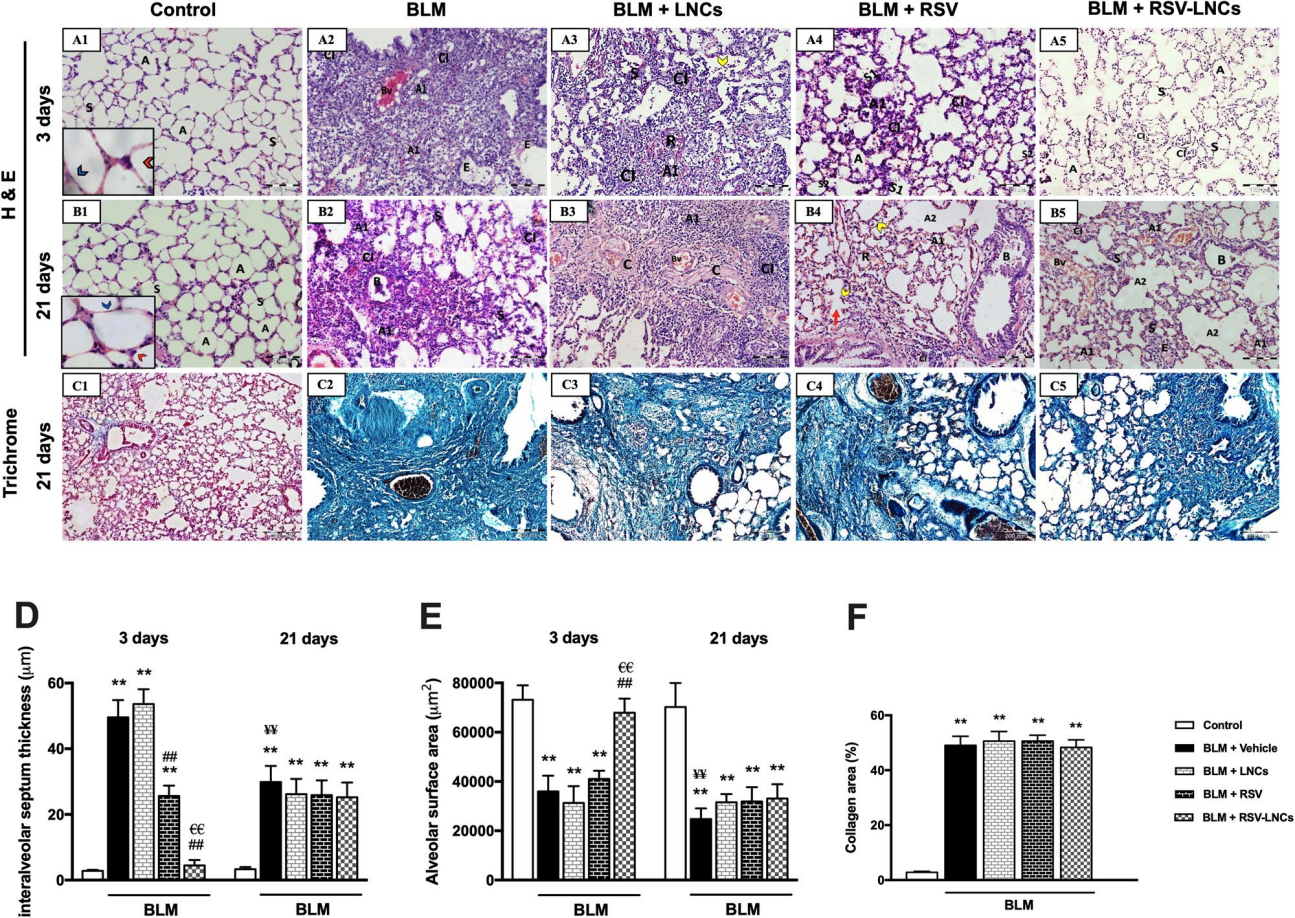
Representative photomicrographs of lung sections of different groups. (A1–A5) Histopathological changes of H&E stained lung sections in animals euthanized 3 days after BLM challenge: (A1) control lung section showing patent alveoli (A) and thin inter-alveolar septa (S). Inset: pneumocytes type I (blue arrowhead) and type II (red arrowhead). (A2) BLM + vehicle group shows mostly collapsed lung alveoli (A1), massive cellular infiltration (CI), congested blood vessel (Bv) and exudate (E). (A3) BLM + LNCs group shows marked alveolar collapse (A1), massive cellular infiltration (CI), extravasation of RBCs (R) and many extruded cells (yellow arrowhead). Marked thickening of the inter-alveolar septa (S) is noted. (A4) BLM + RSV group shows areas of inflammatory cell infiltrate (CI) and narrow alveoli (A1). However, patent alveoli are also present (A). Some septa are thickened (S1), while others appear normal (S2). (A5) BLM + RSV-LNCs group where most alveoli appear patent (A) with intervening apparently normal inter-alveolar septal thickness (S). Few foci of mild cellular infiltration (CI) are noted. (B1–B5) Histopathological changes of H&E stained lung sections in animals euthanized 21 days after BLM challenge: (B1) control lung section showing patent alveoli (A) and thin inter-alveolar septa (S). Inset: pneumocyte type I (blue arrowhead) and type II (red arrowhead). (B2) BLM + vehicle group shows extensive alveolar collapse (A1) surrounded by massive cellular infiltration (CI), thickened septa (S) and exfoliated epithelial lining of a bronchiole (B). (B3) BLM + LNCs group shows wide spread collapse of alveoli (A1) surrounded by massive cellular infiltration (CI). Congested blood vessels (Bv) and obvious deposition of collagen fibers are noted. (B4) BLM + RSV group shows dilated alveoli (A2) and some collapsed alveoli (A1), rupture of septa (red arrow) associated with many extruded cells (yellow arrowhead) within the alveolar lumen. Cellular infiltrate (CI), extravasated RBCs (R) and disruption of epithelial lining of the bronchioles (B) are seen. (B5) BLM + RSV-LNCs group shows collapsed alveoli (A1) alternating with dilated alveoli (A2) and thickened inter-alveolar septa (S). Cellular infiltration (CI), exudate (E), congested blood vessels (Bv) and disrupted bronchiolar epithelium (B) are noted. (C1–C5) Representative photomicrographs of trichrome stained sections of the chronic groups: (C1) control lung shows normal deposition of collagen fibers. (C2) BLM + vehicle, (C3) BLM + LNCs, (C4) BLM + RSV and (C5) BLM + RSV-LNCs groups show excessive deposition of collagen fibers around bronchioles, blood vessels and in the interalveolar septa. (D) Morphometrical analysis of the interalveolar septa thickness and (E) alveolar surface area. (F) Morphometrical analysis of collagen area percentage. Data are presented as the means ± S.D. (*n* = 8/group), and differences between groups at the same time point were analyzed by one-way ANOVA followed, when significant, by Tukey post-hoc test. *p < .05, **p ≤ .001 compared with control; ^#^*p* < . 05, ^##^*p* ≤ .001 compared with BLM + vehicle group; ^€^*p* < .05, ^€€^*p* ≤ .001 compared with BLM + RSV group. Difference of data between acute (day 3) and chronic (day 21) BLM + vehicle groups was analyzed by unpaired t-test, where ^¥^*p* < .05, ^¥¥^*p* ≤ .001.

To assess the effect of RSV-LNCs on the BLM-induced ALI and CPF, histological evaluation was performed at day 3 and day 21, respectively. Control rats showed normal alveolar architecture with thin inter-alveolar septa and patent alveolar spaces (Figure 4A1 and 4B1). Conversely, alveolar structure at day 3 of BLM intoxication (phase of acute lung inflammation) was disrupted. The alveolar septa were markedly thickened (*p* < .001 versus control) as confirmed by morphometric analysis. Extensive cellular infiltration (mainly neutrophils and macrophages) associated with diffuse extravasation of RBCs and marked exudation was noted in the alveolar lumina, many of which were collapsed with significant reduction of the alveolar surface area. Congestion of the pulmonary capillaries and many mitotic figures were detected (Figure 4A2, 4D, and 4E, Figures S1 and S2). Pretreatment with LNCs had no effect on BLM-induced ALI, with persistent distortion of the lung histological structure (Figure 4A3).

Rats pretreated orally with free RSV showed partial amelioration of BLM-induced ALI with alveolar septa significantly thinner than BLM group, but thicker compared to the control (both *p* < .001). Many collapsed alveoli were detected and the alveolar surface area was significantly reduced compared to the control (*p* < .001). Moderate alveolar and interstitial infiltration by inflammatory cells and extravasated RBCs were seen. Macrophages and exudate were occasionally detected (Figure 4A4, 4D, and 4E and Figure S3).

Pretreatment with RSV-LNCs clearly abolished BLM-induced ALI with apparently preserved lung architecture, where most alveoli appeared patent with comparatively normal alveolar surface area and thickness of the inter-alveolar septa (*p* > 0.05 versus control). Foci of mild inflammatory cellular infiltration were hardly observed (Figure 4A5, 4D, and 4E).

**Figure 5.**
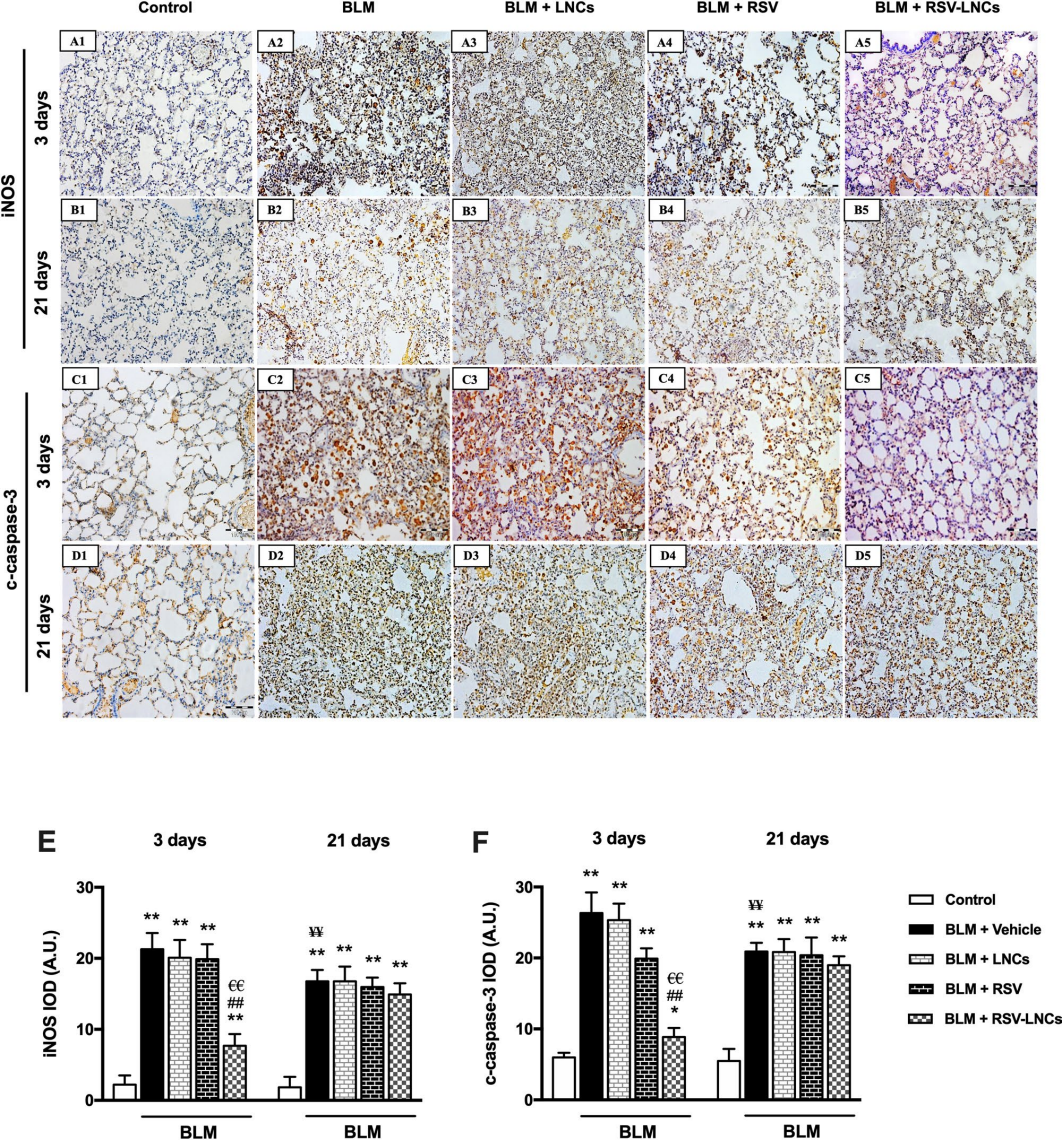
Representative photomicrographs of immunoreactivity of iNOS and c-caspase 3 antibodies. (A1–A5) and (B1–B5) Immunoreactivity to iNOS antibody in lung tissue sections of animals euthanized 3 days and 21 days after BLM challenge, respectively. (C1–C5) and (D1–D5) immunoreactivity to c-caspase 3 antibody in lung tissue sections of animals euthanized 3 days and 21 days after BLM challenge, respectively. The scale bar represents 100 μm. (E, F) Quantitative data obtained after morphometric analysis of the integrated optical density (IOD) of iNOS and c-Caspase 3 expressed as arbitrary units (A.U.). Data are presented as the means ± S.D. Difference between groups (*n* = 8/group) at the same time point was analyzed by one-way ANOVA followed, when significant, by Tukey post-hoc test. *p < .05, **p ≤ .001 compared with control; ^#^*p* < . 05, ^##^*p* ≤ .001 compared with BLM + vehicle group; ^€^*p* < .05, ^€€^*p* ≤ .001 compared with BLM + RSV group. Difference of data between acute (day 3) and chronic (day 21) BLM + vehicle groups was analyzed by unpaired t-test, where ^¥^*p* < .05, ^¥¥^*p* ≤ .001.

In the chronic groups, at day 21 of BLM administration (phase of chronic inflammation and fibrosis), multiple alveoli were collapsed causing marked diminution of gas exchange area, other alveoli were dilated. Significant thickening of the inter-alveolar septa was observed (*p* < .001 versus control group). Extensive cellular infiltration predominantly macrophages and extravasation of RBCs were detected. Bronchiolar epithelium frequently exhibited striking exfoliations (Figure 4B2, 4D, and 4E and Figure S4).

Notably, administration of LNCs, free RSV or RSV-LNCs in the chronic phase could not mitigate inflammation or reverse alveolar disruption. Interalveolar septa thickness and alveolar surface area were not improved (all *p* > 0.05 versus BLM group). Disrupted bronchiolar epithelium, alveolar septa rupture and extensive macrophages infiltration were prominent findings (Figure 4B3–B5, 4D, and 4E, Figures S5 and S6).

Further, to examine the effect of oral free RSV or RSV-LNCs treatment on BLM-induced fibrotic changes, Masson’s trichrome staining for lung tissue sections was performed on the 21st day after BLM challenge. Significant collagen deposition was detected in all groups received BLM (*p* < .001) compared to the control group. All treatments failed to improve pulmonary fibrosis, as no significant difference in collagen area percent was detected between BLM, LNCs, RSV and RSV-LNCs groups (all *p* > 0.05 versus BLM group) (Figure 4C1–C5 and 4F).

### Effect of RSV-LNCs on BLM-induced iNOS and c-caspase 3 expression

The expression of iNOS and c-caspase 3 was assessed using immunohistochemical method to address nitrosative stress and apoptotic signals in the lung tissue, respectively. Results revealed marked increase in iNOS and c-caspase 3 expression at the third day after intratracheal BLM instillation (both *p* < .001), compared to the corresponding control groups that showed minimal expression of both markers. The effect of LNCs on both iNOS and c-caspase 3 was insignificant compared to BLM group (*p* > 0.05). On the other hand, pretreatment with free RSV decreased c-caspase 3 only (*p* < .001), without a significant effect on iNOS expression (*p* > 0.05), whereas RSV-LNCs significantly reduced both iNOS and c-caspase 3 expression in comparison with the BLM group (both *p* < .001) (Figure 5A1–A5, 5C1–C5, 5E, and 5F).

At the 21st day after BLM exposure, despite that iNOS and c-caspase 3 expressions decreased relative to day 3 (*p* < .001 for both), they were significantly higher than corresponding control values (*p* < .001 for both). No significant changes were observed in both markers after RSV, LNCs, RSV-LNCs oral gavage (all *p* > 0.05 versus untreated group), denoting persistent inflammation and apoptosis (Figure 5B1–B5, 5D1–D5, 5E, and 5F).

### Effect of RSV-LNCs on BLM-increased CD68+ immunofluorescence

**Figure 6.**
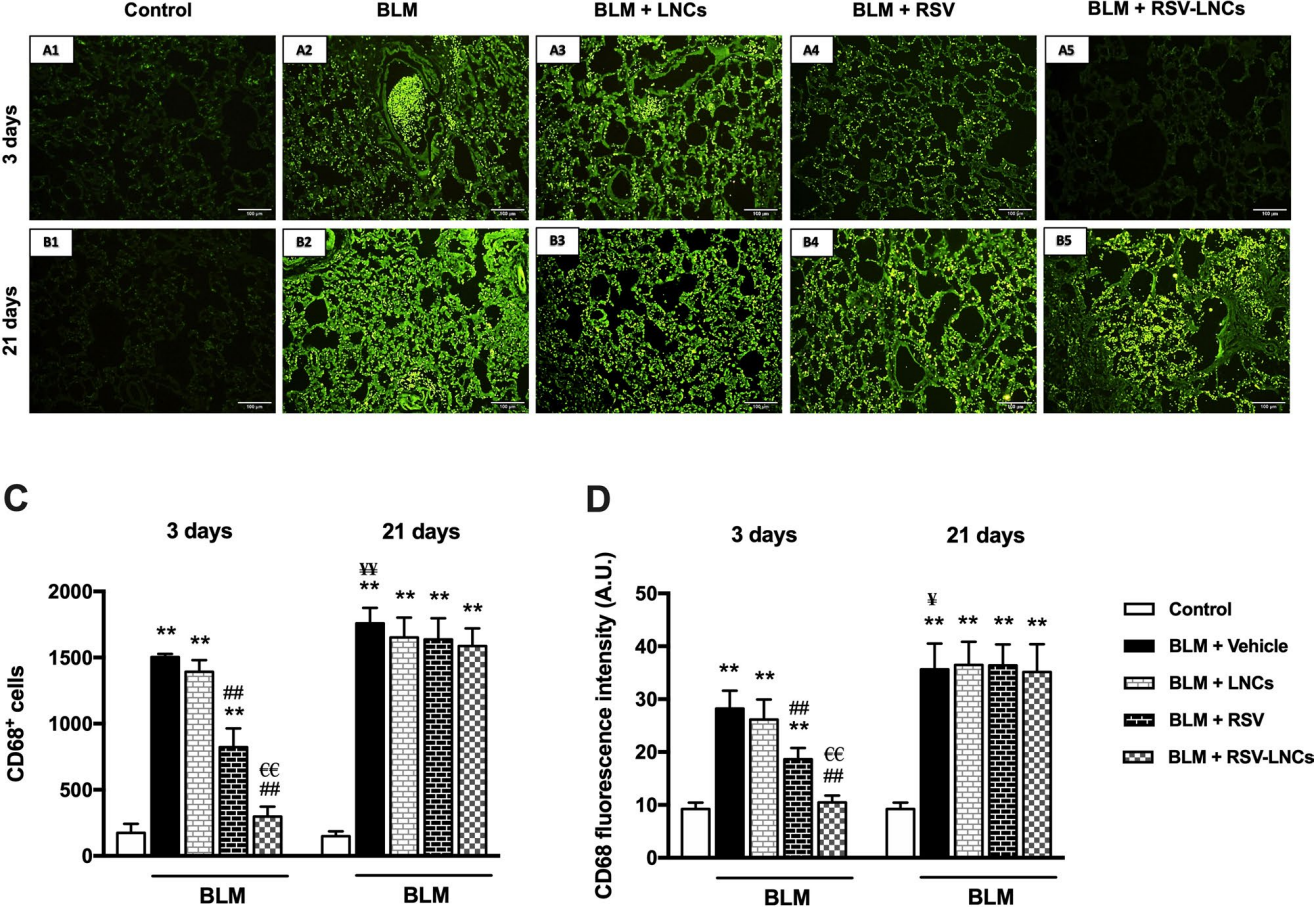
Representative photomicrographs of immunofluorescence of CD68^+^ cells in lung tissue sections of animals euthanized at day 3 (A1–A5) and day 21 (B1–B5) following BLM instillation. (C, D) Quantitative data of morphometric analysis of number of CD68^+^ cells and fluorescence intensity (measured by A.U.). Data are presented as the means ± S.D. Difference between groups (*n* = 8/group) at the same time point was analyzed by one-way ANOVA followed, when significant, by Tukey post-hoc test. *p < .05, **p ≤ .001 compared with control; ^#^*p* < . 05, ^##^*p* ≤ .001 compared with BLM + vehicle group; ^€^*p* < .05, ^€€^*p* ≤ .001 compared with BLM + RSV group. Difference of data between acute (day 3) and chronic (day 21) BLM + vehicle groups was analyzed by unpaired t-test, where ^¥^*p* < .05, ^¥¥^*p* ≤ .001.

Immunofluorescence staining of CD68^+^ (a macrophage marker) revealed that acute BLM intoxication significantly increased macrophage number and activity in lung tissues, compared with control rats on the 3rd day as demonstrated by increased both CD68^+^ cell count and fluorescence intensity (both *p* < .001 versus control). LNCs administration did not show any significant reduction in macrophage number or activity (*p* > 0.05). Both RSV and RSV-LNCs pretreatment counteracted macrophage infiltration leading to a significant decrement in the CD68^+^ cell number and immunoreactivity versus the BLM group (*p* < .001 for all), although levels in RSV group were significantly higher than that of the control and RSV-LNCs groups (all *p* < .001) (Figure 6A1–A5, 6C, and 6D).

Three weeks after intratracheal BLM instillation, the number of CD68^+^ cells and immunofluorescence in pulmonary tissues increased (*p* < .001 and *p* = .003, respectively), as compared with that in the acute phase. Meanwhile, this macrophage infiltration was significantly higher than control values at the same time point (*p* < .001), and was not ameliorated by any treatment applied (all *p* > 0.05 versus BLM group) (Figure 6B1–B5, 6C, and 6D).

### Effect of RSV-LNCs on lung tissue ultrastructure

**Figure 7.**
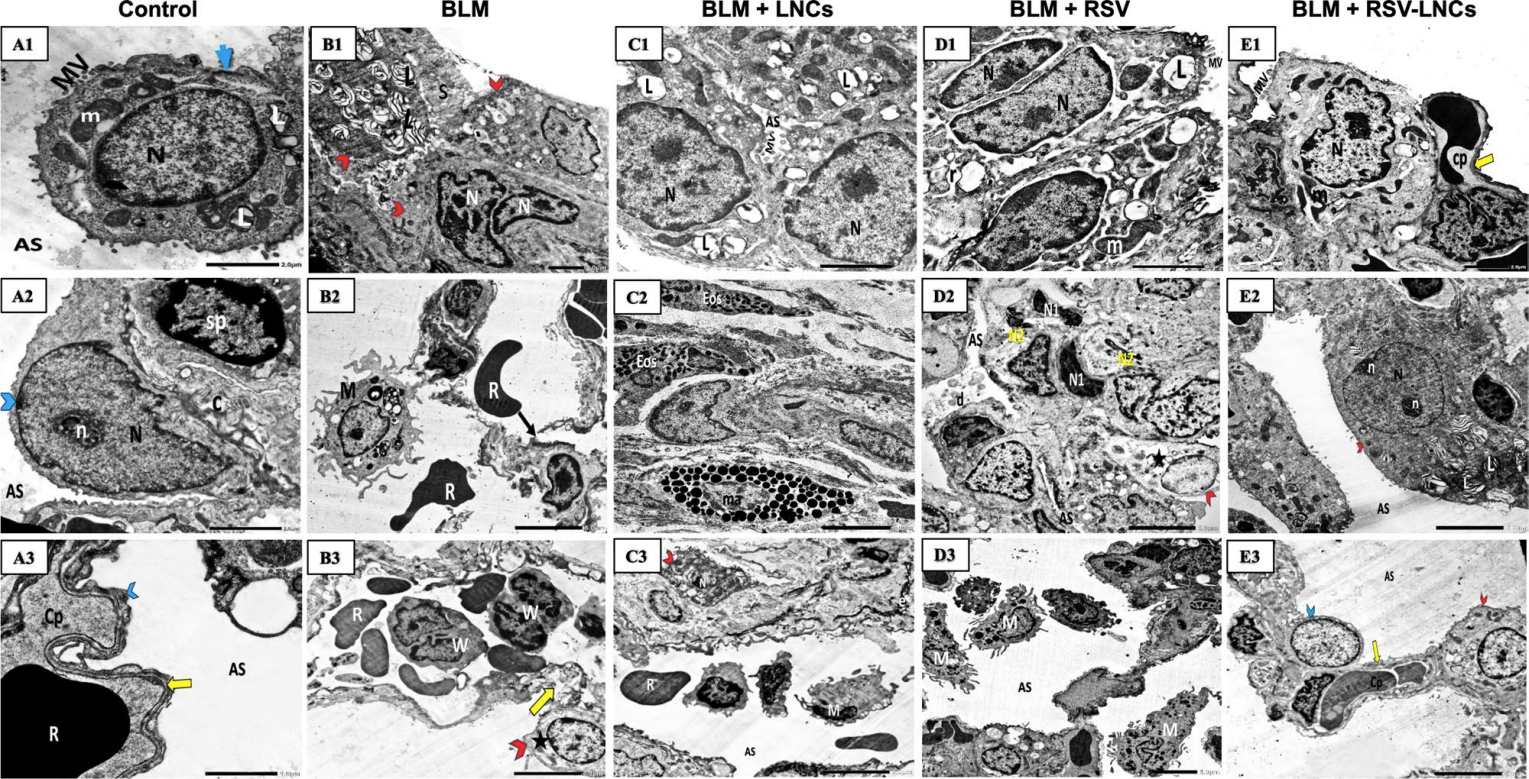
Representative electron photomicrographs of lung tissue in animals euthanized 3 days after BLM challenge. (A1–A3) images of the control group: (A1) A type II pneumocyte exhibits an euchromatic nucleus (N), organized lamellar bodies (L) and multiple mitochondria (m) in their cytoplasm with normal microvillus border (MV). Blue arrow; tight junction. (A2) type I pneumocyte (blue arrowhead) shows an euchromatic nucleus (N) with prominent nucleolus (n) facing the alveolar space (AS). Few amount of collagen (C) together with septal cells (sp) are seen within the inter-alveolar septum. (A3) An intact fused basal lamina (yellow arrow) is demonstrated between pneumocyte type I (blue arrowhead) and a capillary (Cp). R; RBC. (B1–B3) images of the BLM + vehicle group: (B1) shows multiple pneumocyte type II cells (red arrowheads) with increased number of lamellar bodies (L), one cell is binucleated (N). Intra-alveolar secretions (S) are noted in the lumen. (B2) shows an interrupted alveolar wall (black arrow), extravasated irregular RBCs (R) and a macrophage (M) containing multiple lysosomes and extending pseudopodia. (B3) A congested capillary containing multiple RBCs (R) and WBCs (W) with lobulated nuclei. Splitting of the basal lamina (yellow arrow) is seen. Rarefaction (star) of the cytoplasm of a pneumocyte type II (red arrowhead) is also noted. (C1–C3) images of the BLM + LNCs group: (C1) shows multiple pneumocytes type II encroaching upon the alveolar space (AS), they contain euchromatic nuclei (N), vacuolated lamellar bodies (L) and exhibit prominent microvillus border (MV). (C2) shows infiltration by multiple eosinophils (Eos) and a mast cell (Ma). (C3) shows a pneumocyte type II (red arrowhead) with a shrunken heterochromatic nucleus (N), multiple macrophages (M) and extravasated RBCs (R) within the alveolar space (AS). e; elastin fibers. (D1–D3) images of the BLM + RSV group: (D1) shows two active pneumocytes type II, one is binucleated (N), both exhibit dilated perinuclear cisternae together with dilated rER cisternae (r), vacuolated lamellar bodies (L), giant bizarre mitochondria (m) and prominent microvillus border (MV). (D2) shows multiple cells with heterochromatic irregular nuclei (N1), some are fragmented (N2) with marked narrowing of the alveolar spaces (AS) that contain exfoliated debris (d). A Pneumocyte type II (red arrowhead) shows electron lucent cytoplasm (star). (D3) shows multiple macrophages (M) within the alveolar lumen(AS). (E1–E3) images of the BLM + RSV-LNCs group: (E1) shows a type II pneumocyte with an euchromatic nucleus (N), normal microvillus border (MV) and an intact fused basal lamina (yellow arrow). Few bizarre shaped mitochondria (m) are seen. Cp; capillary. (E2) A type II pneumocyte (red arrowhead) with an euchromatic nucleus (N) and two prominent nucleoli (n) associated with increase in the number and lamellation of lamellar bodies (L).AS; alveolar space. (E3) shows preserved alveolar wall lined by normally appearing type I (blue arrowhead) and type II (red arrowhead) pneumocytes and facing patent alveolar spaces (AS). Cp; capillary. Yellow arrow; intact fused basal lamina.

At the ultrastructural level, both control groups revealed patent alveolar spaces lined by normally appearing pneumocytes type I with euchromatic nuclei that were joined by tight junctions to pneumocytes type II that showed euchromatic nuclei and a cytoplasm containing well organized lamellar bodies, mitochondria and normal microvillus border. The basal lamina was seen intact and fused with that of the endothelial cells of the neighboring blood capillary. The inter-alveolar septa were seen containing septal cells with normal content of collagen fibers (Figures 7A1–A3 and 8A1–A3).

Three days following BLM administration, disruption of the alveolar tissue was noted with interrupted alveolar wall and splitted basal lamina. Increased number of type II pneumocytes was evident. Frequently, they appeared binucleated with increased number of lamellar bodies. Other cells exhibited irregular nuclei and rarefied cytoplasm with mitochondria showing disrupted cristae. Alveolar lumina showed fluid exudation and extravasation of RBCs. Infiltration by different types of inflammatory cells including neutrophils and macrophages was noted (Figure 7B1–B3, Figures S7 and S8).

The lung ultrastructure of animals pretreated with LNCs showed disorganization of the alveolar tissue. Alveoli were seen collapsed. Pneumocytes type II were increased in number. Some of them showed shrunken heterochromatic nuclei while others appeared with euchromatic nuclei. Yet, vacuolated lamellar bodies and prominent microvillus border obliterating the alveolar lumen were also detected. Many inflammatory cells in the form of macrophages, eosinophils and mast cells were seen (Figure 7C1–C3).

Rats pretreated orally with free RSV showed some damaged pneumocytes type II with rarefied cytoplasm. Multiple pneumocytes type II were binucleated with dilated perinuclear cisternae and rER cisternae and prominent microvillus border. Some alveolar spaces appeared patent while others were collapsed containing debris. Multiple septal cells showed heterochromatic fragmented nuclei. Furthermore, multiple macrophages were encountered (Figure 7D1–D3).

RSV-LNCs supplementation before BLM significantly preserved alveolar integrity; alveolar spaces were patent and lined by preserved type I pneumocytes lying on an intact basal lamina and apparently normal type II pneumocytes containing normal lamellar bodies and euchromatic nuclei. Occasionally, few pneumocytes type II showed abundant lamellar bodies (Figure 7E1–E3).

**Figure 8.**
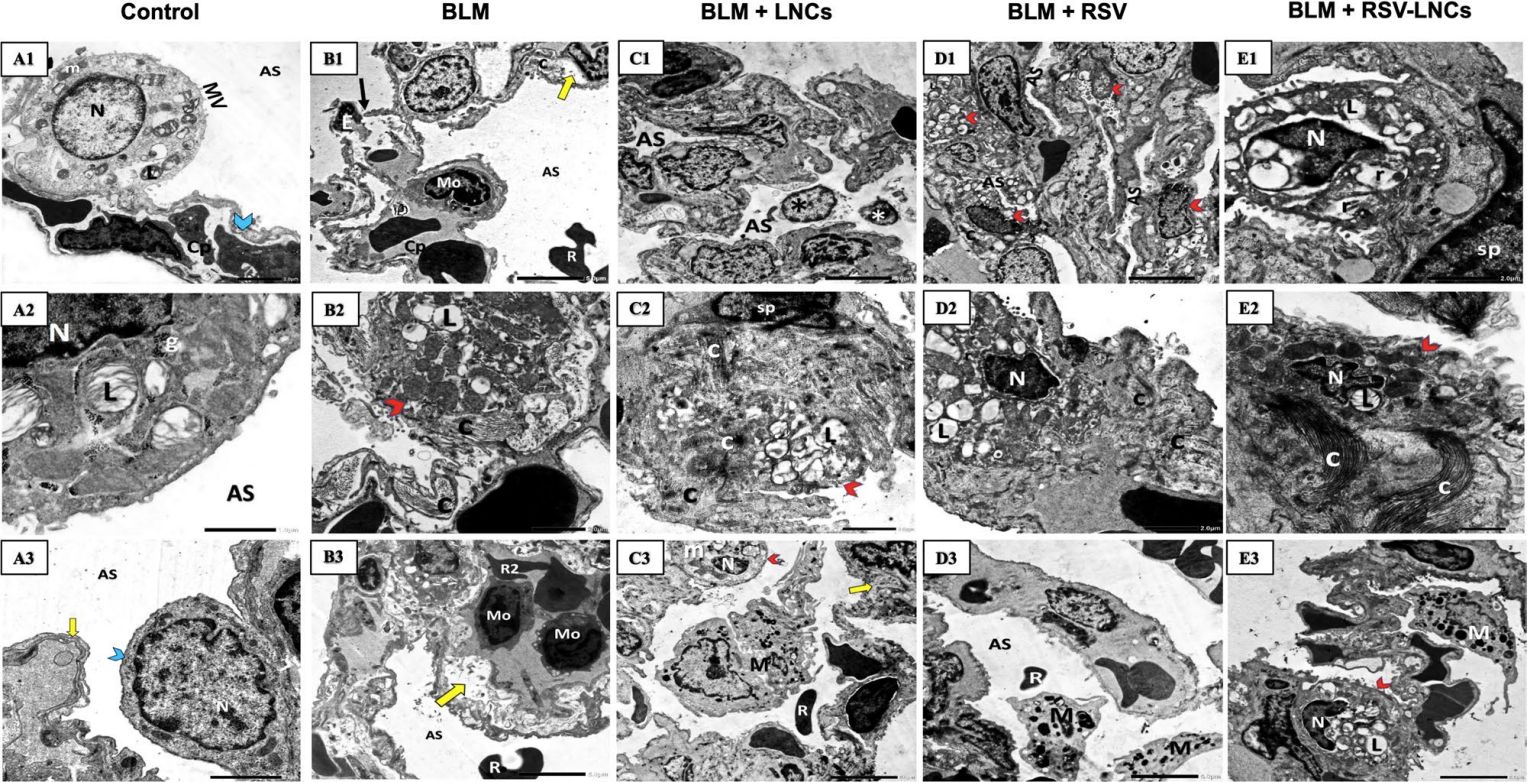
Representative electron photomicrographs of lung tissue in animals euthanized 21 days after BLM challenge. (A1–A3) shows control group type II pneumocyte in A1 that exhibits an euchromatic nucleus (N), organized lamellar bodies (L) and mitochondria (m) in their cytoplasm with normal microvillus border (MV).AS; alveolar space. Cp; capillary. Blue arrowhead; a type I pneumocyte cytoplasm. (A2) High power magnification of a pneumocyte type II shows a nucleus (N) and multiple typically organized lamellar bodies (L) with glycogen (g) accumulation. (A3) A type I pneumocyte (blue arrowhead) shows an euchromatic nucleus (N) and an intact fused basal lamina (yellow arrow). AS; alveolar space. (B1–B3) BLM + vehicle group: (B1) shows disruption of the blood alveolar barrier (black arrow) and splitted basal lamina (yellow arrow). A monocyte (Mo) within the lumen of a capillary (Cp) and irregular extravasated RBCs (R) are seen. E; endothelial cell. AS; alveolar space. (B2) shows a pneumocyte type II (red arrowhead) with vacuolated lamellar bodies (L) with excessive deposition of collagen (C). (B3) A capillary contains multiple monocytes (Mo) and irregular RBCs (R2). Extravasated RBCs (R) within the alveolar space (AS) are seen. Note splitting of the basal lamina (yellow arrow). (C1–C3) BLM + LNCs group (C1) narrow alveolar spaces (AS) containing extruded cells (asterisk). (C2) extensive collagen deposition (C) encroaching upon a pneumocyte type II (red arrowhead) that appears with vacuolated disorganized lamellar bodies (L). sp; septal cell. (C3) A macrophage (M) extending extensive pseudopodia and an extravasated RBC (R) are seen within the alveolar space. A pneumocyte type II (red arrowhead) displaying a shrunken heterochromatic nucleus (N) and fused giant mitochondria(m) is shown. Splitting of the basal lamina (yellow arrow) is noted. (D1–D3) BLM + RSV group: (D1) shows hyperplasia of pneumocytes type II (red arrowheads) and collapsed alveolar spaces (AS). (D2) wide spread collagen deposition(C) impinging on a pneumocyte type II that appears with irregular outlines, a shrunken heterochromatic nucleus (N) and vacuolated lamellar bodies (L). (D3) shows multiple macrophages (M) and an extravasated RBC (R) within the alveolar space (AS). (E1–E3) BLM + RSV-LNCs group: (E1) shows a pneumocyte type II with a shrunken heterochromatic nucleus (N), dilated perinuclear cisternae together with dilated rER cisternae (r), and vacuolated lamellar bodies (L). sp; septal cell. (E2) shows a pneumocyte type II (red arrowhead) with a shrunken irregular heterochromatic nucleus (N) and lamellar bodies (L). Extensive collagen deposition (C) is noted. (E3) A pneumocyte type II (red arrowhead) with a peripheral irregular nucleus (N) and lamellar bodies (L). M; an alveolar macrophage (M).

Twenty-one days after BLM administration, alveolar tissue injury was detected. Blood air barrier interruption, basal lamina splitting and narrow alveoli were evident. Excessive deposition of collagen fibers at multiple sites were seen encroaching upon type II pneumocytes that exhibited irregular outlines and vacuolated lamellar bodies. Other pneumocytes type II appeared with prominent microvillus border, dilated perinuclear cisternae and rER cisternae. Multiple monocytes, plasma cells and extravasated RBCs were also seen. (Figure 8B1–B3, Figures S9 and S10).

In the chronic phase, treatment with LNCs, free RSV or RSV-LNCs was nearly ineffective in alleviating BLM-induced damage in the lung ultrastructure. Collapsed alveolar spaces were observed. Multiple fibroblasts associated with excessive deposition of collagen fibers were exhibited. Many detached cells were seen in the alveolar lumina. Some type II cells exhibited shrunken heterochromatic nuclei and irregular vacuolated lamellar bodies, while others showed dilated perinuclear cisternae together with severe dilatation of rER cisternae and vacuolated lamellar bodies. Proliferated pneumocytes type II were frequently seen. Multiple macrophages with extending pseudopodia were also encountered (Figure 8C1–E3 and Figure S11).

## Discussion

ARDS is a multi-factorial disorder that has a wide spectrum of clinical manifestations and may progress to non-cardiogenic pulmonary edema and fatal respiratory failure. Although supportive care and mechanical ventilation remain the principle management plan, rapid progress in nanomedicine may provide new insights on the pharmacotherapy of ARDS for its remarkable pharmacokinetic and pharmacodynamic enhancing effects (Bian et al., 2021).

It has been reported that BLM-induced lung injury occurs in 40–45% of patients receiving this chemotherapeutic agent with a mortality rate 1–3% (Ro et al., 2020). Intratracheal instillation of BLM is a widely used experimental approach for studying the molecular mechanisms driving lung damage and detecting novel therapeutic strategies (Zhou et al., 2018). In the present experiment, ALI was manifested on the third day following BLM challenge by oxidative damage and inflammatory reaction. This was demonstrated by profound upsurge of leukocytes, mainly neutrophils, and increased SOD activity and MDA levels in the BALF. Moreover, histological examination of BLM-intoxicated lung sections revealed pulmonary congestion and massive recruitment of neutrophils and macrophages as a part of the inflammatory response. The observed alveolar collapse and marked reduction in alveolar surface area may be caused by inability of the alveoli to expand as a result of type II alveolar cell injury with subsequent disruption of surfactant system homeostasis (Steffen et al., 2017). However, cumulative destruction of alveolar elastin by BLM may resulted in distension of some alveoli (Roulet et al., 2012). In addition, thickening of the interalveolar septa was observed, which could be attributed to the development of alveolar edema together with cellular infiltration that significantly added to the thickness of the septa (Steffen et al., 2017). Pulmonary functions were influenced by this damage as demonstrated by lowering the V_T_ and MRV, and increasing the respiratory rate. The bronchioles exhibited disruption of the epithelial lining, further leading to airway dysfunction that was demonstrated by Penh prolongation.

Recent studies demonstrated the success of various nanosystems in the management of different ALI animal models (de Oliveira et al., 2019, 2021; Qiao et al., 2021). Herein, LNCs were selected for the oral delivery of RSV evaluated for the prevention and treatment of acute and chronic BLM-induced lung injury, respectively. LNCs are prepared by a simple scalable low energy solvent free method based on GRAS ingredients (Heurtault et al., 2002). This nanosystem is capable of encapsulating hydrophobic drugs in its oily core with high entrapment efficiency and capability to sustain drug release over a prolonged period (Roger et al., 2011). This allows for the protection of their cargo throughout the journey to the target site. Moreover, bioavailability and activity enhancement of diverse drugs including paclitaxel (Peltier et al., 2006), Sn38^44^, fondaparinux (Ramadan et al., 2011), and miltefosine (Eissa et al., 2015) verify their potential use as oral nanovectors. In this regard, LNCs showed high stability in simulated gastrointestinal fluids (Roger et al., 2009) and intestinal mucus (Groo et al., 2013). Their capability to cross the GIT while retaining their integrity (Eissa et al., 2015; Roger et al., 2017), which is a major challenge facing oral nanomedicine, is also a cornerstone for the success of this nanosystem to target therapeutically relevant distal sites following oral administration.

In the current work, pretreatment with RSV-LNCs blocked BLM-induced ALI with higher efficiency than free RSV. Results demonstrated that RSV loading into LNCs significantly increased its antioxidant, anti-inflammatory and antiapoptotic potency. Functional, biochemical and histological findings were nearly normalized in RSV-LNCs-treated animals, while free RSV only partly mitigated some of these deteriorations. The results of this study are in line with de Oliveira et al., who reported that RSV loading into polymeric nanocapsules improved its potential for the prevention or treatment of lipopolysaccharide-induced ALI (de Oliveira et al., 2019, 2021).

Oral pretreatment with RSV-LNCs effectively ameliorated leukocytosis and neutrophilia in the airway lumen and lung tissues of BLM-intoxicated rats. Decreased neutrophils number can be considered as a direct indicator for mitigation of ALI, because activated neutrophils trigger endothelial damage, basement membrane destruction, and increased vascular permeability leading to pulmonary edema (Dikmen et al., 2021).

A strong association between inflammation and oxidative stress injury involves direct recruitment and activation of neutrophils, which release a substantial number of oxygen and nitrogen free radicals and proteases. The released free radicals promote further lung damage and increase capillary permeability allowing, therefore, cytokines to flow and contribute to more inflammation and edema (Perrone et al., 2012). RSV-LNCs successively interrupted this vicious cycle by normalizing MDA levels and SOD activity.

In this study, iNOS expression was high after 3 days of BLM challenge, while RSV-LNCs pretreatment markedly downregulated iNOS expression, which goes in line with Kececiler-Emir et al. who observed that RSV loading into hybrid silica-PAMAM dendrimer nanoparticles effectively reduced the expression of iNOS in a macrophage cell line induced with lipopolysaccharides (Kececiler-Emir et al., 2021). Indeed, many of the released pro-inflammatory cytokines during ALI boost the expression of iNOS, which generates nitric oxide (NO) that combines with superoxide free radicals forming unstable peroxynitrite. The subsequent formation of potentially damaging byproducts such as hydroxyl and nitrogen dioxide radicals produces nitrosative stress and further contributes to pulmonary injury (Perrone et al., 2012). The alleviation of oxidative and nitrosative imbalance may be the reason for the impressive histopathological improvement by RSV-LNCs, where most alveoli appeared patent with normal alveolar surface area, interalveolar septum thickness, and remarkable absence of cellular infiltration.

On the 3rd day of BLM toxicity, the current research data depicted excessive macrophage infiltration in BALF and increased CD68^+^ cell number and immunofluorescence activity in pulmonary tissues. This increased macrophage number and activity was significantly reduced by RSV-LNCs pretreatment. Cytotoxic macrophages can boost lung injury through several pathways. First, they secrete proinflammatory cytokines such as TNF-α, IL-1β as well as IL-6 that enhance neutrophil recruitment and promote neutrophil leakage from the blood vessels to injured tissue (Deng et al., 2015). Second, they produce damaging reactive oxygen and nitrogen species, as well as potentially harmful bioactive lipids (Laskin et al., 2019). Third, RNA-sequencing demonstrated that macrophages manufacture numerous pro-fibrotic chemokines, such as CCL2 and CCL24, which recruit fibroblasts and are positively linked with the progression and severity of fibrosis in BLM-induced lung injury (Cheng et al., 2021). Furthermore, a recent research demonstrated that IL-6 enhances fibrosis by promoting chronic inflammation and stimulating TGFβ pathway, the most robust profibrotic cytokine reported (Epstein Shochet et al., 2020). Thus, by suppressing macrophages number and activity, and decreasing IL-6 levels, RSV-LNCs could effectively prevent progression of ARDS to irreversible pulmonary fibrosis.

Apoptosis is acknowledged to play a major role in BLM-induced lung injury (Zaki et al., 2021). Herein, BLM toxicity was associated with a significantly high c-caspase 3 immunopositivity. The caspase system could be stimulated by elevated level of TNF-α or lipid peroxidation. Moreover, oxidative stress disrupts mitochondrial membrane permeability and triggers the release of proapoptotic proteins like cytochrome c, which further induces caspase-3 activation (Alanazi et al., 2020). RSV-LNCs reverted the BLM-induced increase in c-caspase 3 by subsiding the inflammation and oxidative stress. This anti-apoptotic effect may be an additional mechanism contributing to the protection of pulmonary tissues against BLM toxicity.

Previous research demonstrated that type II pneumocytes expansion may be the most sensitive histopathological sign of alveolitis, and the quantity of type II pneumocytes reflects the severity of alveolar injury (Honda et al., 2003). Therefore, it was interesting to shed light on pulmonary tissue ultrastructural changes using TEM. In the present work, lung tissues of acute BLM toxicity group demonstrated pneumocytes type II hyperplasia that might be a compensatory mechanism to the observed extensive injury to type I pneumocytes, which are highly sensitive to damage. As previously reported, type II pneumocytes immediately begin a burst of proliferation to replace the destroyed cells and reestablish the alveolar epithelium’s integrity (Tian et al., 2020; Wang et al., 2020). In addition, multiple type II pneumocytes were seen hyperactive with euchromatic nuclei, prominent nucleoli, excessive lamellar bodies and prominent microvillus border in a trial to compensate surfactant dysfunction caused by the injury. Some alveolar cells exhibited morphological features of degeneration such as rarified cytoplasm, vacuolated lamellar bodies and shrunken heterochromatic nuclei. Demonstration of some bizarre shaped giant mitochondria may be considered as an adaptation mechanism to free radical exposure (Shami et al., 2021). Similar ultrastructural apoptotic findings were documented in previous research (Deng et al., 2015). RSV-LNCs pretreatment remarkably reverted many ultrastructural abnormalities, where most alveoli appeared patent without any remarkable inflammatory cells seen in the alveolar lumina, which were separated by preserved inter-alveolar septa. The alveoli were lined by apparently normal type I and type II pneumocytes.

The antioxidant, anti-inflammatory and anti-apoptotic effects observed in acute RSV-LNCs group with subsequent preservation of lung tissue were positively reflected on pulmonary functions, where the V_T_, breathing frequency, MRV as well as the inspiratory flow rate were all comparable to normal. In addition, the normal Penh indicates absence of airway dysfunction.

Previous studies revealed that, if not properly treated, BLM-injured lung tissues progress to chronic interstitial lung fibrosis (Baek et al., 2020). In line, after 21 days of BLM exposure, rats showed persistent lung tissue injury, oxidative stress and inflammation although MDA level, SOD activity, iNOS and c-caspase 3 were lower than in the acute phase. On the contrary, IL-6 and CD68^+^ immunofluorescence was markedly increased. Histopathological examination showed wide areas of collapsed alveoli which markedly decreased gas exchange area together with thickened interalveolar septum, cellular infiltration and bronchiolar epithelium exfoliation. This chronic inflammation stimulated epithelial expansion and gave rise to a marked hyperplasia of type II alveolar cells. In line, it was previously reported that IL-1β produced by macrophages promotes abnormal mitosis of type II pneumocytes and inhibits their differentiation to alveolar type I pneumocytes (Vannella & Wynn, 2017). Remarkably, severe fibrosis was evident in trichrome stained and TEM sections. As a consequence of these pathological alterations, WBP results showed that pulmonary functions were markedly deteriorated, where V_T_ and MRV were very restricted, and Penh was prolonged denoting increased resistance to air flow by the excess fibrosis.

Previous studies demonstrated an anti-fibrotic effect for high doses of RSV administered immediately after disease induction and before establishment of chronic inflammation (Conte et al., 2015; Ding et al., 2019; Azmoonfar et al., 2020). This study may be the first to address the effect of delayed administration of RSV or RSV-LNCs on prohibiting BLM-induced chronic inflammation and fibrosis. Unfortunately, the tested dose of RSV, either free or loaded on LNCs, was not capable of improving the histopathologic changes observed in the chronic phase. The distorted oxidative state and the chronic inflammatory process were not resolved with continuous increase in IL-6 along with extensive macrophage infiltration and activity. All these resulted in tissue apoptosis exacerbation and enhanced fibroblasts accumulation. Accordingly, collagen deposition was remarkable and pulmonary functions were not improved. Although RSV was previously shown to inhibit fibrosis through restoration of redox balance and amelioration of oxidative damage (Cheresh et al., 2021), herein, the antioxidant effect of RSV was not detectable in the chronic phase of BLM toxicity. These data are partially in line with Azmoonfar et al., who reported that RSV was unable to mitigate inflammation and cellular infiltration in radiation-induced pulmonary fibrosis model (Azmoonfar et al., 2020). These contradictory findings regarding the antifibrotic effect of RSV could be attributed to the time of administration and sacrifice following disease induction and also to the dose used, where Wang et al., previously reported failure of low RSV dose to alleviate chronic inflammation and fibrosis in BLM model of pulmonary injury (Wang et al., 2021).

## Conclusion

The present study emphasized that pretreatment with RSV loaded into LNCs is more effective in the prophylaxis against acute pneumonitis than free RSV. RSV-LNCs exerted an evident antioxidant, anti-inflammatory and antiapoptotic effects, and preserved pulmonary tissue architecture and function. The chronic inflammation and fibrosis, however, were not ameliorated by the tested dose of RSV or RSV-LNCs. Further studies are required to optimize the dose and duration of RSV-LNCs for appropriate therapeutic effect, especially in the chronic phase.

## Supplementary Material

Supplemental Material
