# Supplementary material for: Histopathological, physiological and biochemical assessment of resveratrol nanocapsules efficacy in bleomycin-induced acute and chronic lung injury in rats
Source: Drug Deliv. 2022 Aug 9;29(1):2592–608. doi: 10.1080/10717544.2022.2105445 (PMC9373765; doi:10.1080/10717544.2022.2105445)
Supplement: Supplemental Material [file IDRD_A_2105445_SM3149.docx]

**Supplementary material**

**Histopathological, physiological and biochemical assessment of resveratrol nanocapsules efficacy in bleomycin-induced acute and chronic lung injury in rats**

**Neama M. Albanawany^a^, Doaa M. Samy^b^ , Noha Zahran^a^, Riham M. El-Moslemany^c^, Shefaa MF. Elsawy^a^, Maha W. Abou Nazel^a^**

**^a^** Department of Histology and Cell Biology, Faculty of Medicine, Alexandria University, Alexandria, Egypt.

**^b^** Department of Medical Physiology, Faculty of Medicine, Alexandria University, Alexandria, Egypt.

**^c^** Department of Pharmaceutics, Faculty of Pharmacy, Alexandria University,21521 Alexandria, Egypt


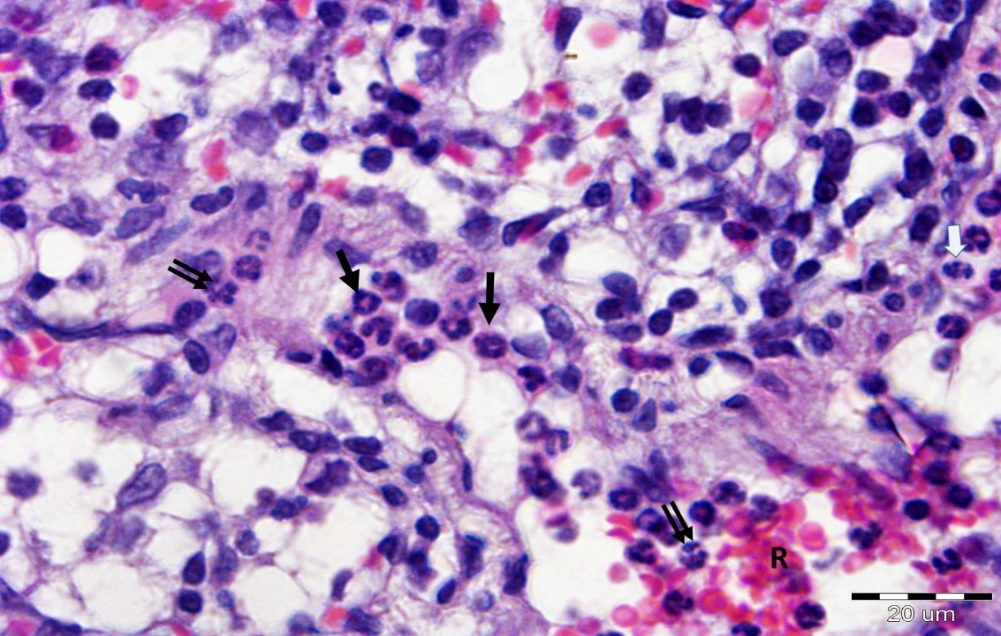


**Figure (S1):** Photomicrograph of acute BLM group lung section showing extensive cellular infiltration mainly in the form of neutrophils (black arrow), some of which appear hyper-segmented (double arrows). Extravasated RBCs (R) and occasional mitotic cells (white arrow) are noted.


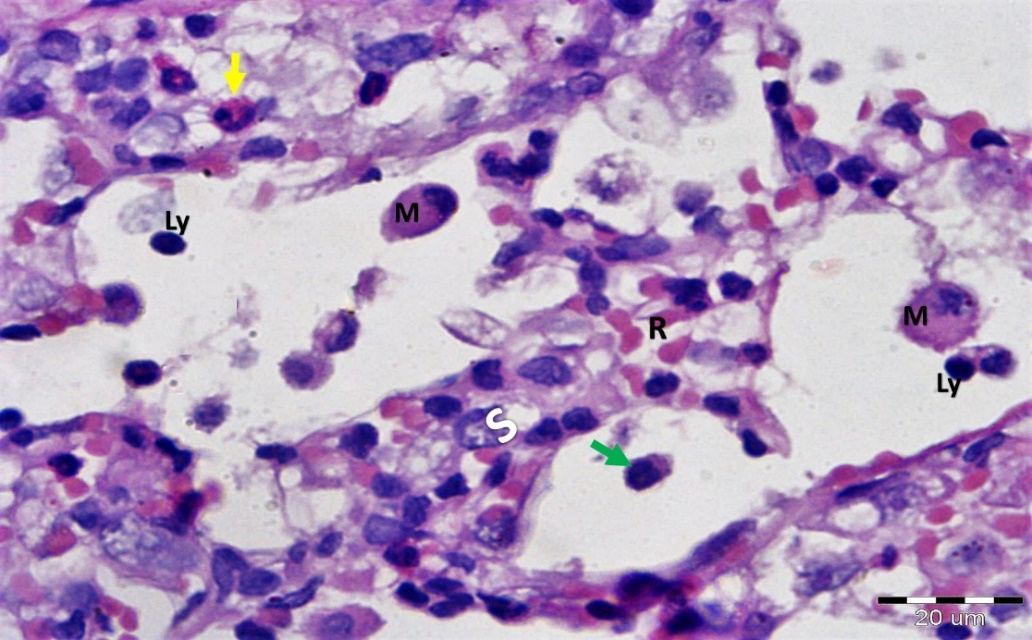


**Figure (S2):** Photomicrograph of acute BLM group lung section showing marked thickening of the inter-alveolar septa (S). Multiple macrophages (M) mixed with lymphocytes (Ly) and basophils (green arrow) are seen within the alveolar lumen. Some extravasated RBCs (R) and eosinophils (yellow arrow) are noted.


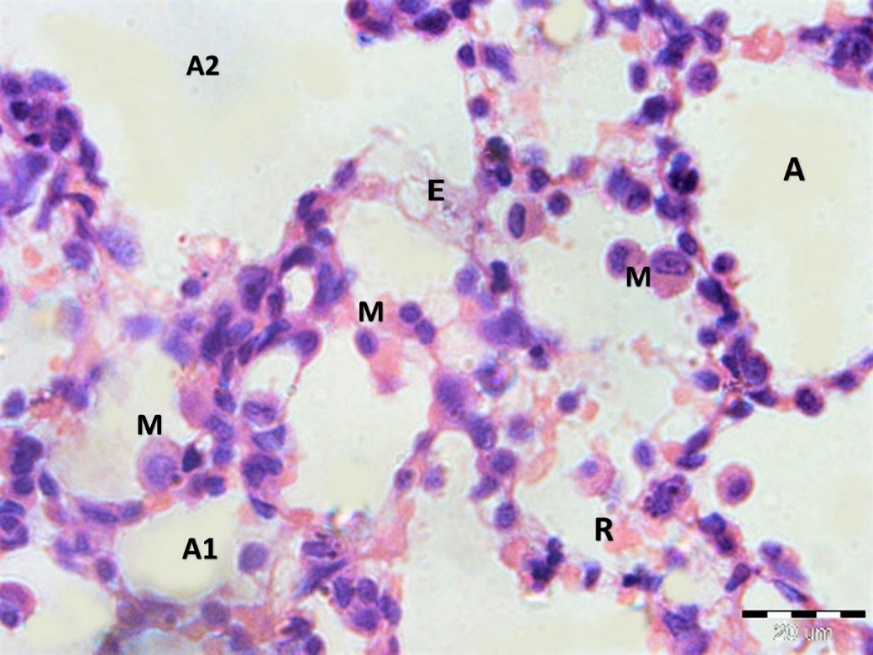


**Figure (S3):** Photomicrograph of the acute group pretreated with RSV lung section showing narrow alveoli (A1) alternating with dilated (A2) and patent alveoli (A). Alveolar macrophages (M), extravasated RBCs (R) and exudate (E) are noted.


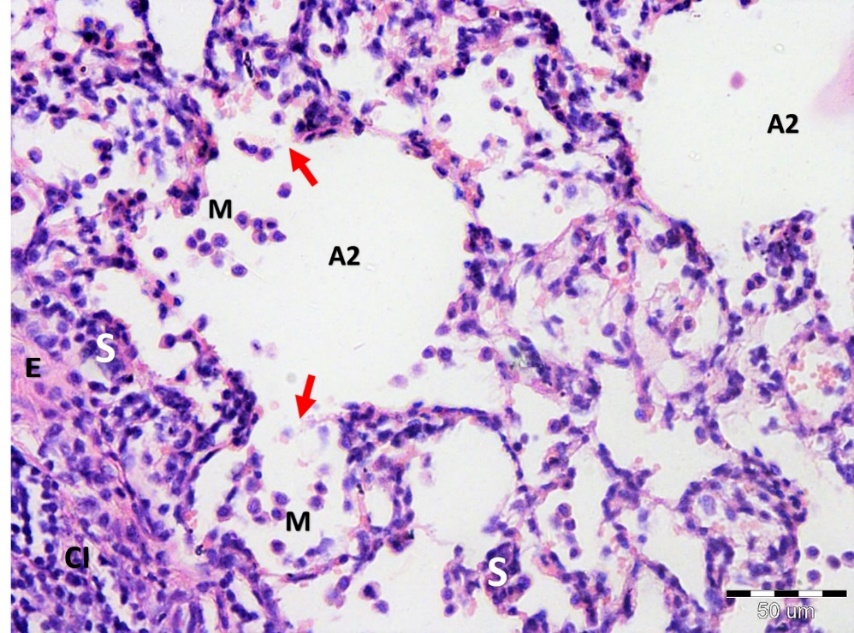


**Figure (S4):** Photomicrograph of chronic BLM + vehicle group lung section, showing dilated alveoli (A2) and disrupted septa (red arrow). Huge amount of macrophages (M) are seen within the alveolar lumina. Cellular infiltration (CI), exudate (E) and thickened septa (S) are noted.


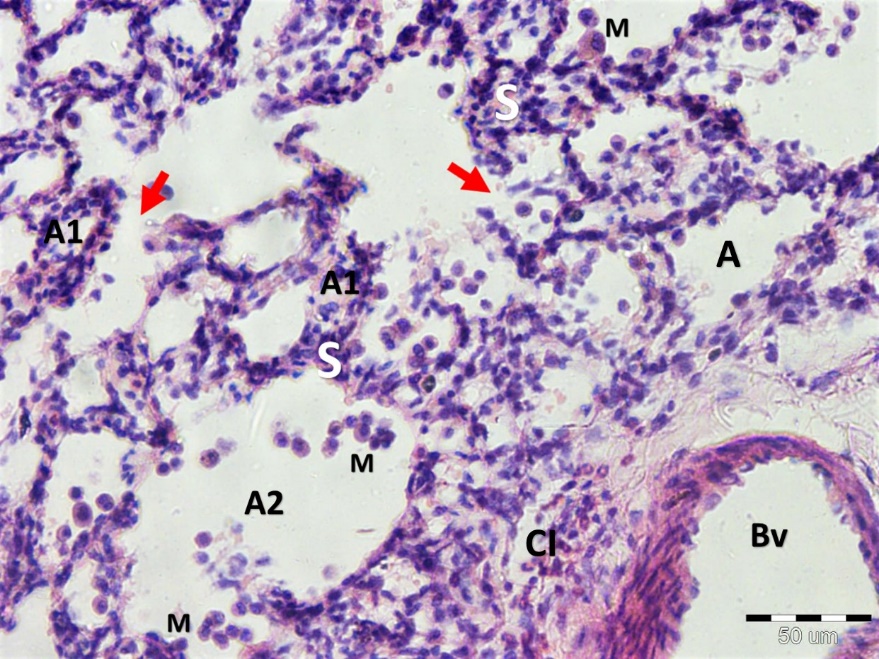


**Figure (S5):** Photomicrograph of chronic BLM + RSV group lung section showing dilated alveoli (A2) alternating with collapsed alveoli (A1).). The inter-alveolar septum (S) is disrupted (red arrow) at multiple sites and thickened (S) at others. Heavy infiltration by alveolar macrophages (M) is noted. Perivascular cellular infiltration (CI) around the blood vessel (Bv) that exhibits thickened wall is seen. A; patent alveoli.


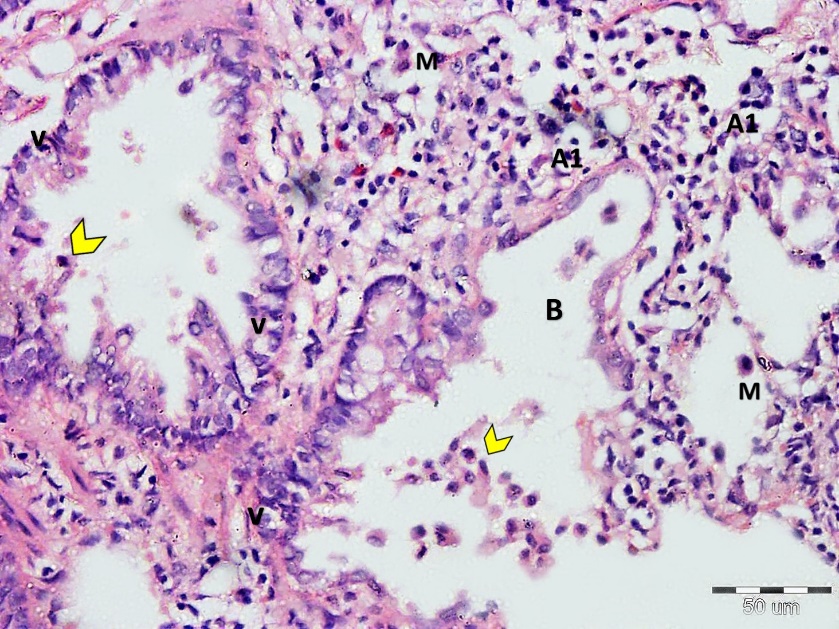


**Figure (S6):** Photomicrograph of chronic BLM + RSV-LNCs group lung section showing collapsed alveoli (A1). Severe disruption and exfoliation of the epithelium of a bronchiole (B) with many extruded cells (yellow arrowhead) within the lumen and vacuolation (v) of the bronchiole lining cells are noted. M; macrophages.


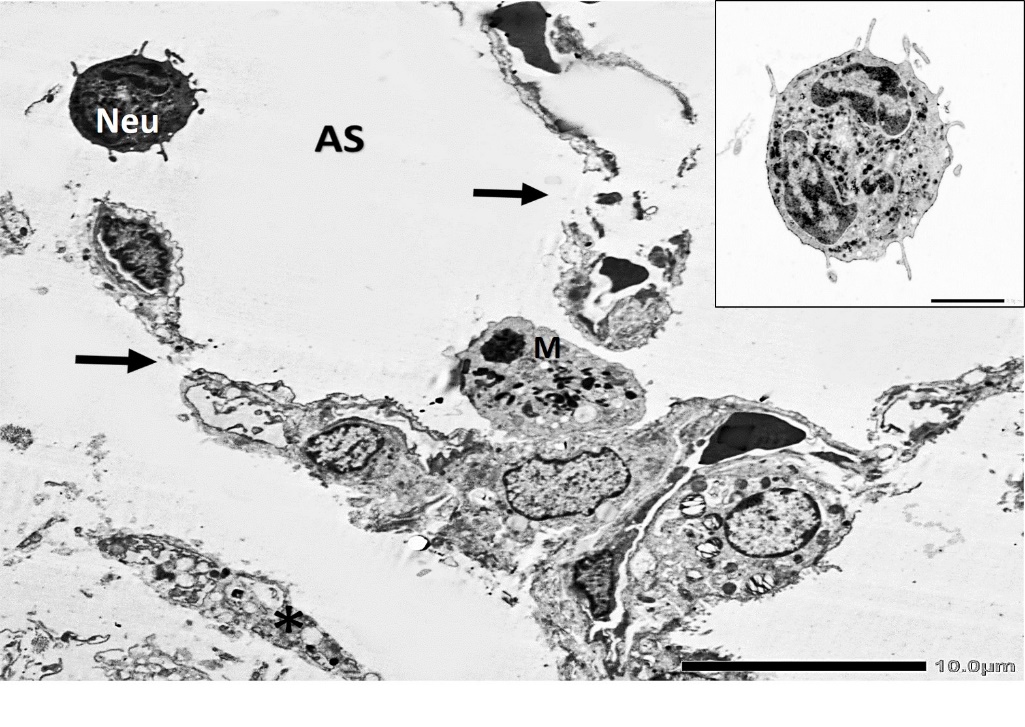


**Figure (S7):** An electron photomicrograph of acute BLM intoxicated group showing interrupted alveolar wall and blood air barrier (black arrows) and luminal infiltration by neutrophils (Neu) and macrophages (M). Intra-alveolar degenerated cell (asterisk) is also seen. AS; alveolar space.


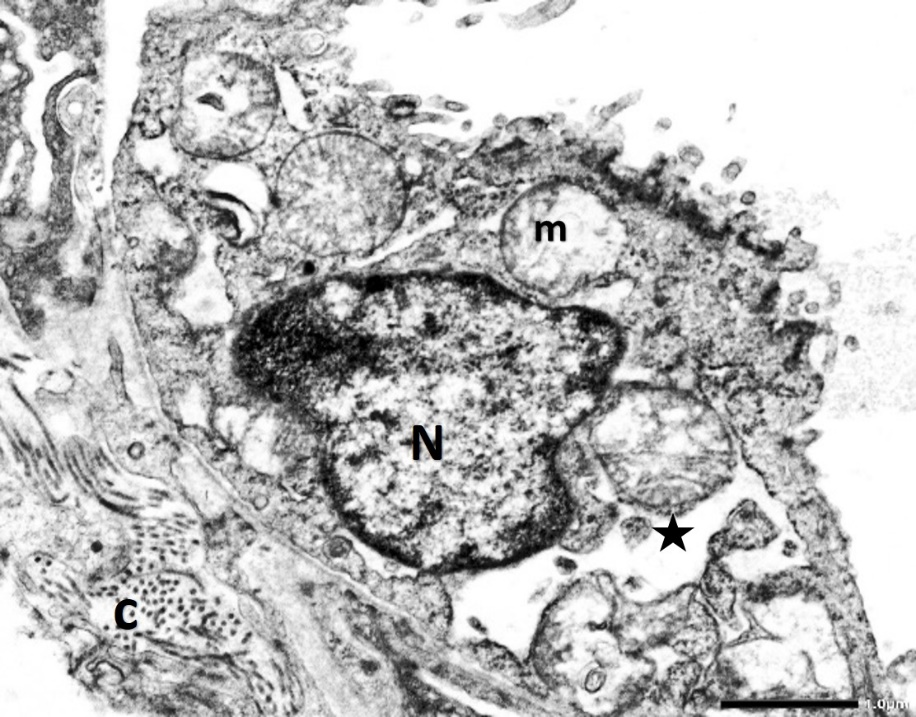


**Figure (S8):** An electron photomicrograph of acute BLM intoxicated group showing a pneumocyte type II with electron lucent cytoplasm (black star**),** disrupted mitochondrial cristae (m) and an irregular nucleus (N). C; collagen.


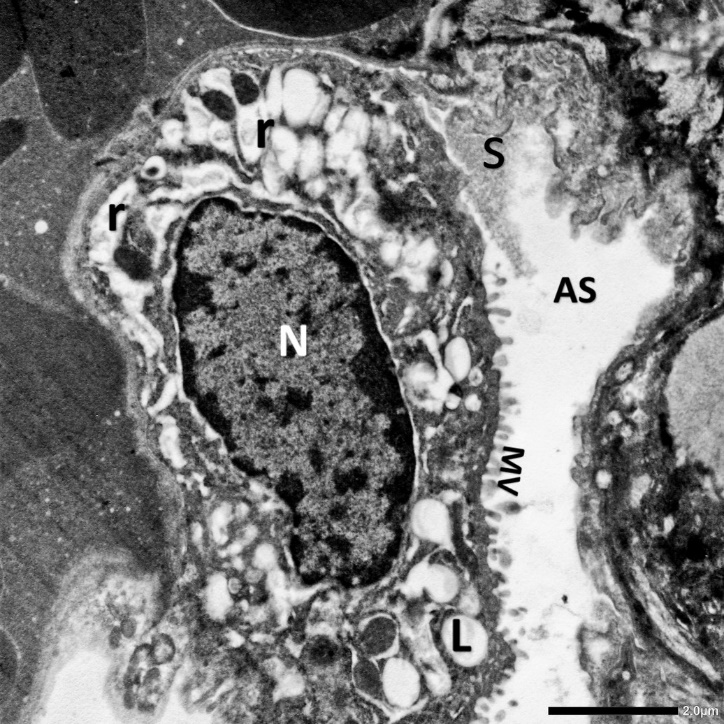


**Figure (S9):** An electron photomicrograph of chronic BLM + vehicle group showing a pneumocyte type II containing an euchromatic nucleus (N) with dilated perinuclear cisternae, markedly dilated cisternae of rER (r), vacuolated lamellar bodies (L) and prominent microvillus border (MV). Intra-alveolar secretions (s) are noted. AS; alveolar space.


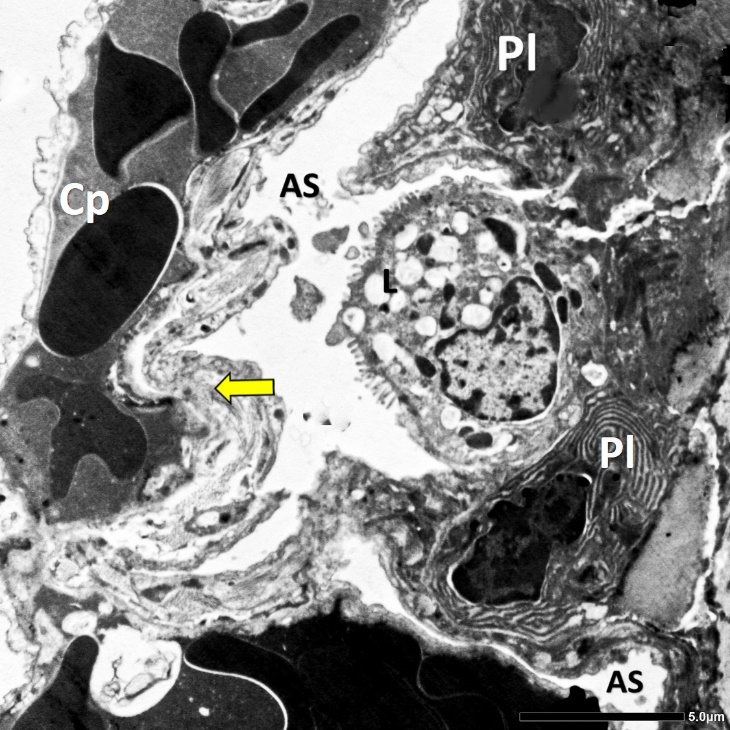


**Figure (S10):** An electron photomicrograph of chronic BLM + vehicle group showing splitted basal lamina (yellow arrow) and narrow alveolar spaces (AS). Plasma cell infiltration (Pl) is observed. Cp; capillary.


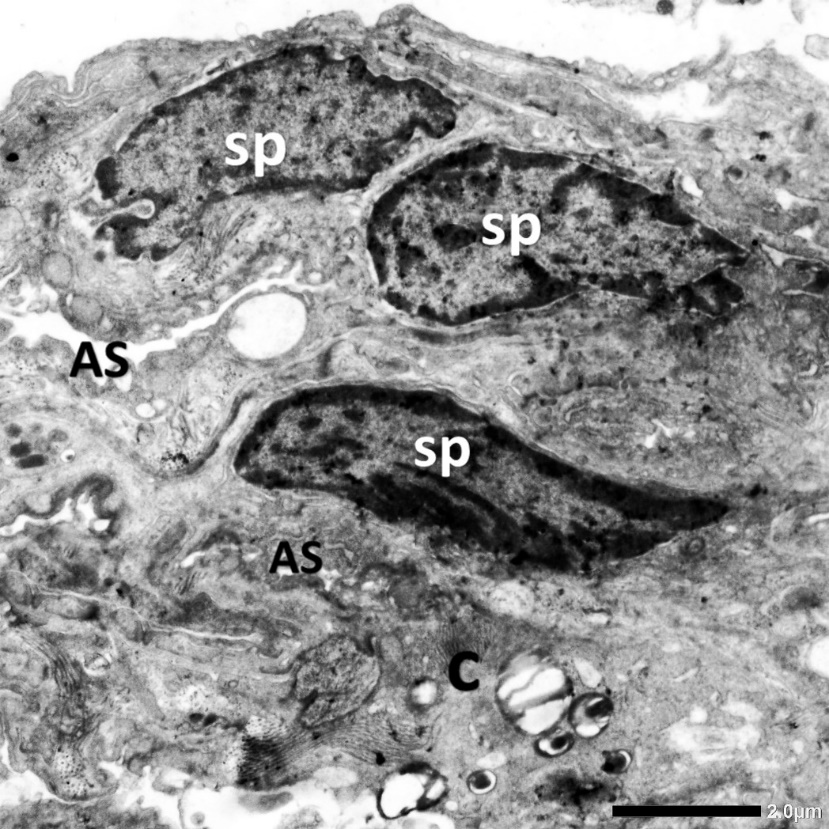


**Figure (S11):** An electron photomicrograph of chronic BLM + LNCs group showing thickened inter-alveolar septa by multiple septal cells (sp) and extensive collagen deposition (c) together with obliterated alveolar spaces (AS).
